# Sequencing and characterization of *Helcococcus ovis*: a comprehensive comparative genomic analysis of virulence

**DOI:** 10.1186/s12864-023-09581-1

**Published:** 2023-08-30

**Authors:** Federico Cunha, Segundo Casaro, Kristi L. Jones, Rafael S. Bisinotto, Subhashinie Kariyawasam, Mary B. Brown, Klibs N. Galvão

**Affiliations:** 1 Department of Large Animal Clinical Sciences, University of Florida College of Veterinary Medicine, Gainesville, FL USA; 2 Department of Animal Sciences, University of Florida College of Agriculture and Life Sciences, Gainesville, FL USA; 3 Department of Comparative, Diagnostic and Population Medicine, University of Florida College of Veterinary Medicine, Gainesville, FL USA; 4 Department of Infectious Diseases and Immunology, University of Florida College of Veterinary Medicine, Gainesville, FL USA; 5 D. H. Barron Reproductive and Perinatal Biology Research Program, University of Florida, Gainesville, FL USA

**Keywords:** *Helcococcus ovis*, Dairy cow, Uterine disease, Nanopore, Complete genome, Hybrid genome assembly

## Abstract

**Background:**

*Helcococcus ovis (H. ovis)* is an emerging bacterial pathogen that commonly causes opportunistic respiratory, mammary, and uterine infections across mammalian hosts. This study applied long- and short-read whole genome sequencing technologies to identify virulence factors in five *H. ovis* isolates with low, medium, and high virulence phenotypes.

**Results:**

The resulting assemblies contained one circular chromosome ranging from 1,744,566 to 1,850,083 bp in length and had a mean GC content of 27.6%. Phylogenetic and nucleotide identity analyses found low virulence strain KG38 to be part of a clade that forms an outgroup apart from the rest of the *H. ovis* taxon. Assembling the first complete genomes of the species revealed major genomic rearrangements in KG38. One to six prophage regions were identified in each genome. A novel pathogenicity island was found exclusively in the two high virulence strains (KG37 and KG104), along with two hypothetical transmembrane proteins designated as putative VFs. Finally, three zinc ABC transporters and three Type-II/IV secretion systems were identified as possible virulence determinants in this species. The low virulence strain KG38 has fewer intact paralogs of these operons in its genome compared to the higher virulence isolates, which strongly suggests a role in virulence. This strain is also missing four putative virulence factors (VFs) found in other isolates associated with adherence (collagen adhesin precursor), immune evasion (choline-binding protein A and a PspA-like hypothetical protein) and cell wall synthesis (glycerol-3-phosphate cytidylyltransferase).

**Conclusions:**

In this study, we assembled reference-quality complete genomes for five *H. ovis* strains to identify putative virulence factors. Phylogenetic analyses of *H. ovis* isolates revealed the presence of a clade representing a potentially novel species within the genus *Helcococcus*. A novel pathogenicity island and two hypothetical transmembrane proteins were found exclusively in high-virulence strains. The identification of Zinc ABC transporters and Type-II/IV secretion systems as possible virulence determinants, along with the differences in operon content between the low and high virulence isolates, strongly suggests they also play a role in the bacterium’s pathogenicity. Taken together, these findings are a valuable first step toward deciphering the pathogenesis of *H. ovis* infections.

**Supplementary Information:**

The online version contains supplementary material available at 10.1186/s12864-023-09581-1.

## Background

*Helcococcus ovis* (*H. ovis*) is a Gram-positive, facultative anaerobic, multi-host animal pathogen that often goes undetected due to the specific conditions required for its isolation [1–3]. The bacterium is a recurrent feature in mixed infections in domestic ruminant hosts, including mastitis [4], pneumonia septic arthritis [5], and metritis [6]. Although *H. ovis* is known to rely on pyridoxal provided by co-occurring bacteria to grow in-vitro, its ability to cause infections independently has been demonstrated [4, 5, 7]. Bovine isolates of *H. ovis* alone can cause mastitis and synergize with *Trueperella pyogenes* (*T. pyogenes*) to inflict more severe lesions in the mammary glands of murine infection models [4]. In dairy calves with *Mycoplasma bovis* (*M. bovis*) pneumonia, co-infecting *H. ovis* can cause septic arthritis and produce areas of lung necrosis alongside the caseous lesions seen in *M. bovis* infections [5, 8]. In dairy cows (*Bos taurus)*, a higher abundance of *H. ovis* in the uterus has been associated with metritis [6], but its role in the pathogenesis of the disease remains unclear due to the complexity of the uterine microbiota [9, 10]. Although *H. ovis’* capacity to cause infection and interact with other bacteria is well-documented, the molecular mechanisms of infection and virulence determinants remain unexplored.

*H. ovis* was first identified as a distinct species closely related to *Helcococcus kunzii* (*H. kunzii*) by comparative 16S rRNA gene sequencing and biochemical testing [1]. Since then, there have been dozens of sequenced 16S rRNA genes of *H. ovis* isolates from animal infections [3, 4, 7]*.* Amplicons of *H. ovis* 16 s rRNA have also been identified in association with diseases in humans such as peritoneal effusion, vaginal infections, and endometrial neoplasms [11]. An isolate representing the only verified *H. ovis* infection in humans was recovered from a human artificial eye and was classified as *H. ovis* Tongji strain, primarily based on having a 16 s rRNA identity of 98.9% with other *H. ovis* isolates [12]. However, its atypical metabolic profile for the species and its phylogenetic position in a very closely related outgroup within the *Helcococcus* genus remain unexplained [12]. There are no complete genomes of the bacterium publicly available, and the type strain (CCUG 37441 T) genome has not yet been sequenced. We have previously published short-read draft genome sequences of *H. ovis* isolates that are highly fragmented and have no virulence data associated with them [13]. This dearth of available genetic information has made analyses of phylogenetic relationships among isolates across hosts and infection sites unreliable, as they have been limited to 16S rRNA genes only.

We have established and applied an invertebrate infection model to *H. ovis* from the uterus of dairy cows and identified five isolates with varying degrees of virulence [14]. Four of these isolates originate from the uterus of dairy cows with metritis and were shown to be moderately (KG36, KG104) to highly virulent (KG37, KG106). The fifth isolate (KG38) originates from the uterus of a healthy cow and displayed significantly attenuated virulence. The established relative virulence of these isolates can be used to identify potential virulence determinants in the species to better understand the molecular mechanisms of *H. ovis* infection.

Complete genome assemblies are used to study the structural variations of haploid genomes with more accuracy than what is achievable through draft assemblies [15]. In bacteria, structural variations can result from horizontal transfer of mobile genetic elements which can lead to profound virulence phenotype alterations [16, 17]. These mobile genetic elements are often encountered as genomic islands within the bacterial chromosome and contain accessory genes that provide selective advantages like rare metabolic pathways, antimicrobial resistance genes, and virulence factors [18]. Although no virulence-associated genomic islands have been identified in Helcococci, species-specific pathogenicity islands are a well-documented phenomenon in other Gram-positive organisms like Staphylococcus aureus [19]. Recent advances in long-read DNA sequencing and hybrid long-read-first assembly methods have given us the ability to produce near-perfect complete bacterial genomes [20]. Producing reference-quality complete genomes of strains with established virulence capabilities is a necessary first step towards understanding the molecular mechanisms of *H. ovis* infection. In this study, we sequenced and analyzed the complete genomes of these five *H. ovis* isolates to explore their phylogenetic relationships, genetic diversity, and virulence factors.

## Results

We compared the genomic features of five *H. ovis* isolates originating from the uterus of dairy cows (*Bos taurus)*. These isolates have been classified into three virulence categories, high (KG37 and KG106), medium (KG 36 and KG104), and low (KG38) [14]. Detailed isolate information is listed in Supplemental Table 1. By combining the complete sequenced genomes of these isolates with their previously determined relative virulence categories, we gained a better understanding of the phylogenetic relationships and putative virulence determinants of this emerging animal pathogen.

### Genome sequencing and features

The genomes of these five isolates were sequenced and assembled using both Oxford Nanopore Technologies (ONT) and Illumina platforms, which resulted in the assembly of complete reference-quality genomes. After quality filtering, the resulting ONT reads had a mean coverage of the *H. ovis* genome (≈1.8 Mbp) of 96.4x, ranging from 54 × to 205x. The overall mean read length was 8,751 bp with means ranging from 6,663 bp to 10,710 bp. The overall mean read quality (PHRED + 33) was 12.4, ranging from 12.3 to 12.8. The filtered Illumina reads resulted in a mean coverage of the *H. ovis* genome of 950x, ranging from 531 × to 1568x. The mean percentage of base pairs with a quality greater than 30 was 93.5%, ranging from 92.0% to 96.5%. Detailed sequencing read information is presented in Supplemental Table 2.

All five sequenced genomes were successfully assembled into single circular chromosomes with a mean genome length of 1.80 Mbp. *H. ovis* KG36 had the shortest genome (≈1.74 Mbp) and KG38 the longest (≈1.85 Mbp). No plasmids were found in any of the genomes. The overall 27.6% GC content of these genomes agrees with that of previously reported *H. ovis* genomes [13]. All assemblies have completeness benchmarking scores higher than 95.0%, indicative of high-quality genomes. Genome annotation identified a mean of 1,097 proteins with identified functional assignment and 602 hypothetical proteins (HP) per genome. Genome statistics and genomic features are listed in Table 1.

**Table 1:** Genomic features of *H. ovis* complete genome assemblies

**Isolate**	**Size (Mbp)**	**GC%**	**CDS**	**Annotated Proteins**	**Hypothetical Proteins**	**CDS ratio**	**rRNA**	**tRNA**
KG36	1.77	27.47	1657	1068	589	0.93	4	33
KG37	1.74	27.40	1630	1080	550	0.93	4	33
KG38	1.85	27.88	1778	1127	651	0.96	4	33
KG104	1.79	27.56	1691	1087	604	0.94	4	33
KG106	1.85	27.69	1730	1125	605	0.94	4	33

### Phylogeny and average nucleotide identity

**Fig. 1:**
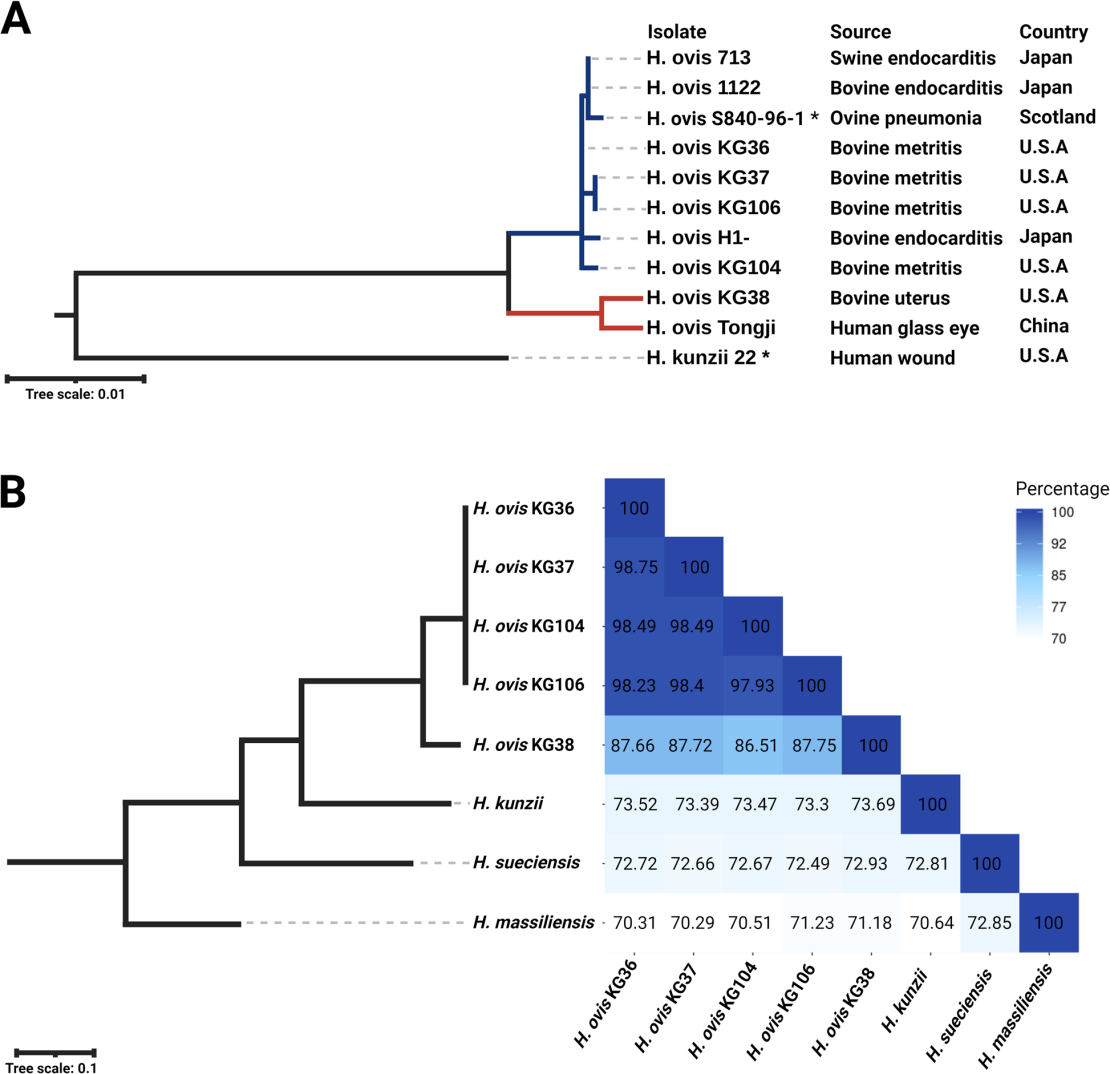
Panel **A** shows a maximum-likelihood single-gene phylogenetic tree of *H. ovis* 16s rRNA sequences on the NCBI nucleotide database. The sequences used were selected to represent isolates from across host species and geographical locations. *H. kunzii,* the type species for the *Helcococcus* genus is included to contextualize the position of *H. ovis* outgroups. The red-colored branch is the clade shared by *H. ovis* KG38 and the Tongji strain. The blue-colored branch marks the clade containing the majority of the *H. ovis taxon*. The type strain of each species is marked by an asterisk (*) next to the isolate label. Panel **B** shows a maximum-likelihood phylogenetic tree and heat-map of pairwise ANI values for each isolate in this study and the type strains of each species within the *Helcococcus* genus

To explore the genetic relationships between the isolates in this study in the context of the *Helcococcus* genus, a single-gene phylogenetic tree was constructed using *H. ovis* 16s rRNA sequences on the National Center for Biotechnology Information (NCBI) nucleotide database (Fig. 1A). The *H. ovis* sequences used were selected to include isolates from across host species and geographical locations. *H. kunzii,* the type species for the *Helcococcus* genus, was included in the analysis to contextualize the position of any possible *H. ovis* outgroups. The resulting phylogenetic tree showed *H. ovis* is divided into two distinct clades. *H. ovis* KG38 belongs to the clade shared only by the Tongji strain, which originates from a human host in China [12]. The rest of the isolates in this study share a different clade with isolates from varied animal hosts across Europe, Asia, and America. To have more robust evidence for the existence of distinct clades within the *H. ovis* taxon, we constructed a phylogenetic tree based on 445 single-copy genes found in all species of the *Helcococcus* genus (Fig. 1B). The resulting phylogenetic tree confirms the findings of the single-gene phylogenetic analysis in which KG38 is in a closely related, but separate, clade to the other isolates from this study.

Pairwise average nucleotide identities (ANIb) were calculated using the isolates that were included in the multi-locus phylogenetic tree plus two other *H. ovis* genomes (KG-39 and KG-40) available on NCBI (Supplemental Table 3). KG38 has ANIb values ranging from 86.5% to 87.8% when compared to all other *H. ovis* isolates, which is below the generally accepted 95% threshold for bacterial species delineation [21]. However, the ANIb values of KG38 compared to the type strains of other *Helcococcus* species were much lower: 73.7% with *H. kunzii*, 72.9% with *Helcococcus sueciensis (H. sueciensis)*, and 71.18% with *Helcococcus massiliensis (H. massiliensis*). These ANIb values show KG38 is as dissimilar to other non-*H. ovis* species as they are to each other (Fig. 1B). All other *H. ovis* species have ANIs greater than 97.0% with each other, including the two *H. ovis* genomes (KG-39 and KG-40) retrieved from NCBI. These genetic distances suggest KG38 may represent a distinct strain within the *H. ovis* taxon or a novel species that is closely related to *H. ovis.*

### Genomic structural comparison

**Fig. 2:**
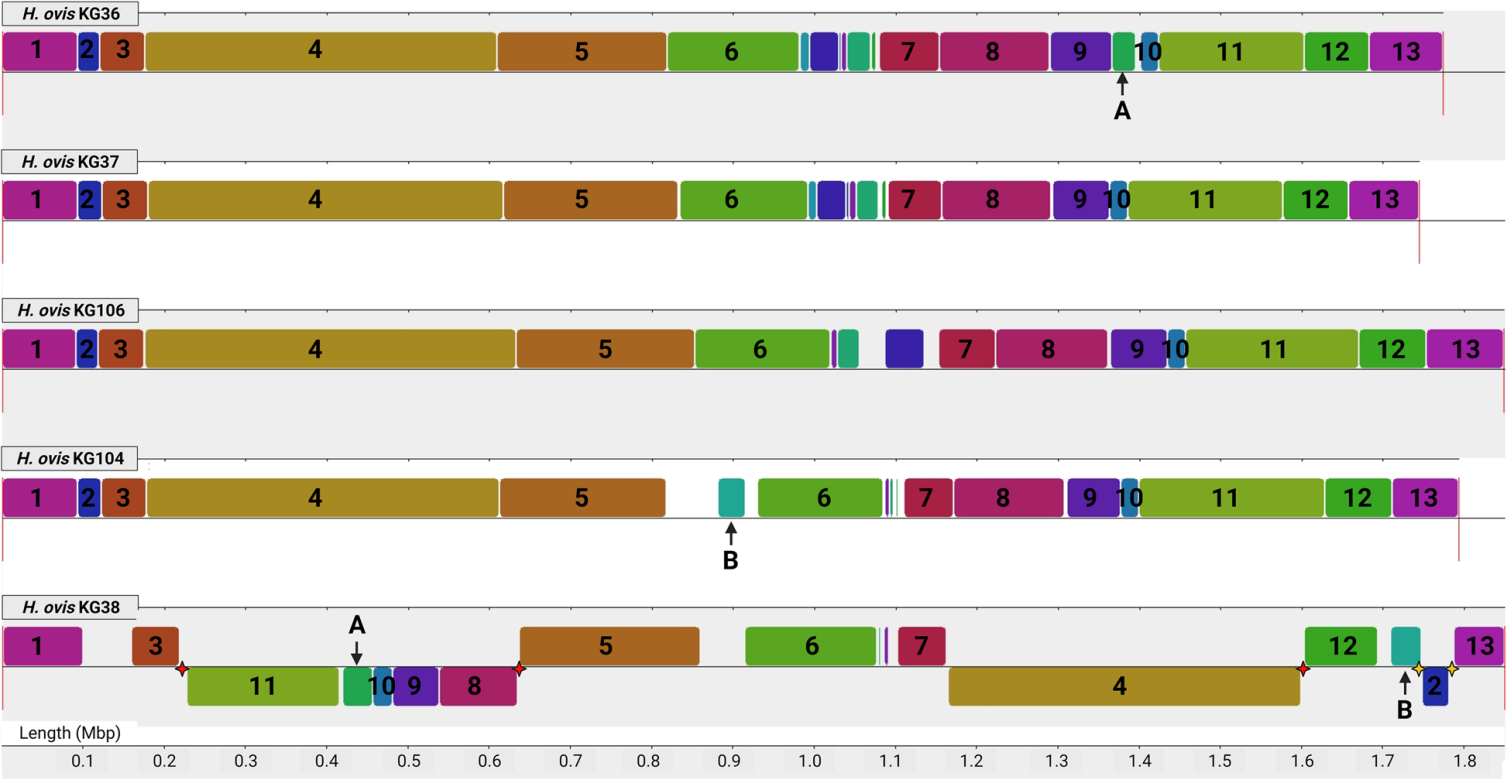
Multiple sequence alignments of *H. ovis* genomes using MAUVE. The thirteen LCBs shared among all these genomes are numbered 1 to 13. LCBs only shared between KG38 and one other genome are labeled A and B. Red stars represent transposable elements. Yellow stars represent prophage integrases

Having assembled high quality complete circular genomes enabled us to evaluate their organizational structures and identify patterns of chromosomal rearrangement that may affect virulence phenotypes. Multiple sequence alignments of all five *H. ovis* genomes showed a high degree of synteny with minimal structural differences between isolates KG36, KG37, KG104, and KG106. A similarly high degree of synteny was found in KG38 but with several major rearrangements in the genome. As shown in Fig. 2, all five genomes share thirteen locally collinear blocks (LCBs) containing conserved sequence elements that make up the majority of each of the genomes. The LCBs of isolates KG36, KG37, KG104, and KG106 are arranged in identical order and only have significant heterologous regions separating LCBs 5, 6, and 7. These heterogenous areas with very short LCBs are also found in the same region of the KG38 genome. However, the position and structure of large LCBs shared with the other *H. ovis* genomes suggest major translocation events occurred in the genome of low-virulence KG38.

All five isolates have LCB 2 containing all the components of an ABC transporter of undetermined function and the only genomic copy of *clpB*, which codes for a chaperone protein that plays a crucial role in heat shock survival in bacteria [22]. In KG38, LCB 2 has a weight of 29,344 bp, is flanked by phage mobile elements, and is inverted and translocated within a heterologous region downstream from LCB 12. This heterologous region also includes a prophage associated LCB B, which is present only in KG104 and KG38. Secondly, LCB 4, which has a weight of 439,309 bp, is also inverted and translocated to the region between LCBs 7 and 12 in KG38. This LCB is flanked by a transposable element downstream, but none are found upstream. The strains KG106 and KG37 have a collagen adhesin precursor gene spanning the junction of LCBs 3 and 4, and KG104 and KG36 have an alcohol dehydrogenase gene spanning the same region. KG38 however does not contain any of these genes in that position, indicating there may have been a deletion event to account for the absence of a transposable element upstream from LCB 4. Finally, a region spanning LCBs 8 through 11 is flanked by transposable elements in both direction and is inverted and translocated to the region between LCBs 3 and 5 in KG38. LCB A, a prophage region with a weight of 13,264 bp, is present within this region in KG38 and is exclusively shared by KG36, located between LCBs 9 and 10.

As shown in Supplemental Fig. 1, KG36, KG104, KG37, and KG106 have four large LCBs in identical order and a region of structural heterogeneity downstream from LCB 14. Only isolates KG36 and KG37 have LCB C, but they also share LCB D with KG106. These LCBs are not present in KG 104, which has repeat regions and CRISPR-associated proteins in the region where these LCBs are found in the other genomes. This area of relative heterogeneity across *H. ovis* genomes is dominated by repeats, spacers, and proteins associated with the organism’s CRISPR/Cas systems.

### Mobile genetic elements

Putative prophage sequences in the *H. ovis* genomes are shown in Supplemental Fig. 2 and summarized in Supplemental Table 4. The low virulence isolate KG38 has more putative prophage regions than any other isolate, including three intact and three incomplete prophage regions that in total account for 10% of the bacterium’s genome. The numbered phage regions 1, 4, and 5 of KG38 coincide with gaps in the MAUVE alignment and are not shared by any other *H. ovis* genomes. Prophage regions homologous to region 2 and 6 in KG38 are also present in KG36 and KG104, respectively. Putative prophage region 2 is the only one adjacent to a translocated LCB on KG38, suggesting that although the density of prophage sequences is high in this genome, they are likely not the main driver of the structural rearrangements that differentiate KG38 from the other isolates.

Isolates KG36, KG104, and KG106 share an incomplete conserved putative prophage region (Region 1), which includes two subunits of a heterodimeric efflux transporter protein. Although it was not flagged by PHASTER, elements of this putative prophage were found in KG37 and KG38 through manual searches. The putative prophage is structurally identical in KG37, but it is truncated by another mobile genetic element carrying *tetA* and *tetB* antimicrobial resistance genes. KG38 contains half of the genes found in this prophage region, including the two subunits of a heterodimeric efflux transporter protein. However, it is missing the downstream integrase, two HPs, two phage-like proteins and the terminase present in the other four genomes (Supplemental Fig. 3).

Integrative and conjugative elements (ICEs) containing Type IV secretion systems (T4SS) were identified in every *H. ovis* genome. One of the highly virulent strains, KG106, has two T4SS-carrying ICE regions. One of these regions is located within a putative prophage region identified by PHASTER. The remaining isolates all contained only one T4SS-encoding ICE. All the ICEfinder-identified mobile elements and their associations with putative prophage regions are listed in Supplemental Table 5.

### Protein families and virulence determinants

**Fig. 3:**
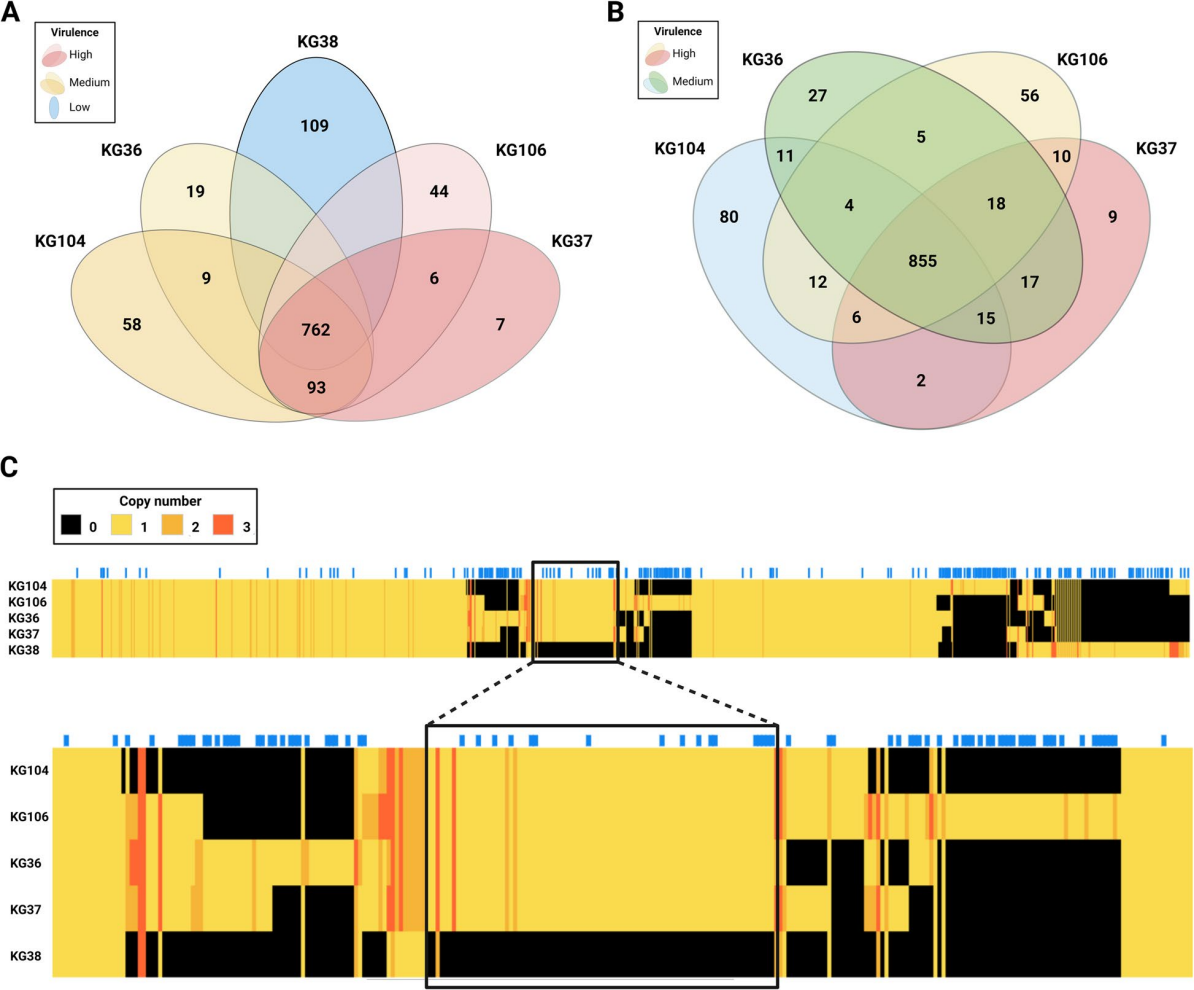
Venn diagrams and heat map comparing 1,236 protein families shared by all the *H. ovis* isolates. The Venn diagram in Panel **A** includes all five isolates in this study. Panel **B** compares only high and medium virulence strains. Panel **C** is a heatmap providing an overview of the distribution of protein families across the genomes. Genomes are on the vertical axis and Protein Families are listed along the horizontal axis. The blue ticks along the top of the horizontal axis mark protein families comprised of phage associated hypothetical proteins. The red box highlights the cluster of protein families with assigned functions present in all genomes except KG38. These protein families represent the portion of core proteome of the high and medium virulence strains which are missing from KG38. The color of each cell represents how many proteins from a family are present in each genome (black = 0, bright yellow = 1, dark yellow = 2, dark orange ≥ 3)

As shown in Fig. 3, 762 of the 1,236 protein families identified are shared by all the *H. ovis* isolates. KG38 has 109 unique protein families not found in any of the other genomes, and there are 236 protein families present in at least one of the high and medium virulence isolates that are not shared by KG38. The heat map of the protein families reveals most of the proteins that account for these differences are HPs associated with prophage regions. However, there is a large area at the center of the heat map with a high density of protein families with assigned functions shared by all isolates except KG38 and which possibly contains the coding sequence (CDS) responsible for the virulence differences between the two groups. The 93 protein families found in this region of the heatmap represent the protein families of the medium and high virulence strains core proteome that is absent from low virulence strain KG38. Finally, when compared in the absence of KG38, isolates KG36, KG37, KG104, and KG106 have 855 protein families in common. There are six protein families exclusively present in both high virulence strains (KG37 and KG106) and nine present only in the medium virulence strain (KG36 and KG104).

### Putative virulence factors exclusive to high virulence strains

**Fig. 4:**
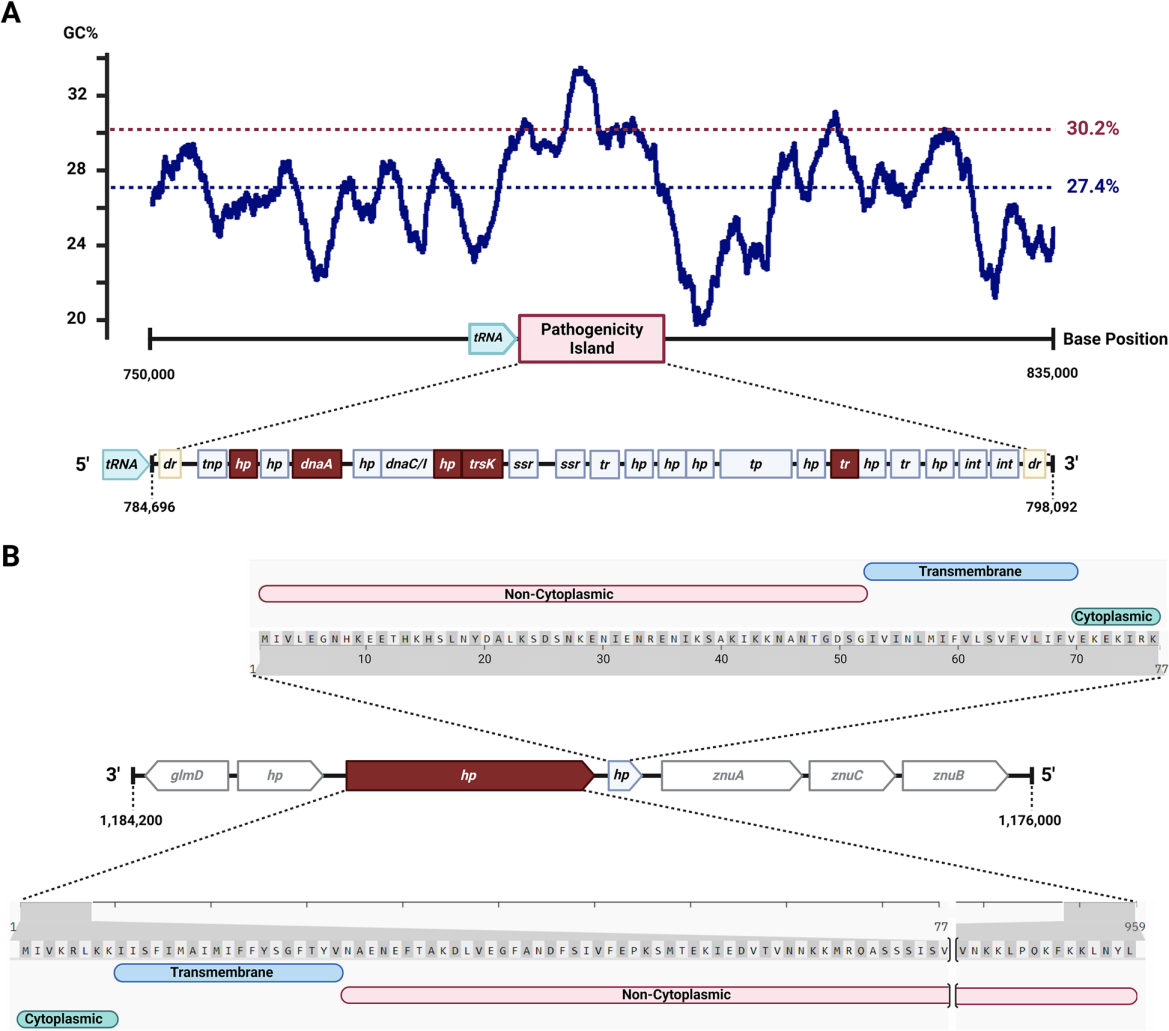
Genomic contexts of putative virulence factors found exclusively on high virulence *H. ovis* isolates (KG37 and KG106). Panel **A** shows the GC content of the putative pathogenicity island (red dotted line) is higher than the mean chromosomal GC content (blue dotted line). CDS colored in red represent proteins that had positive hits in the VFDB. Panel **B** shows the CDS and protein domains for two hypothetical membrane proteins upstream from a ZnuABC operon on high virulence *H. ovis* isolates

The member proteins of four of the six protein families exclusive to the high virulence isolates were found near each other, located downstream from a Lysyl-tRNA gene and within a 12,484 bp region flanked by 465 bp direct repeats. The entire genomic island (GI) is absent from the low and medium virulence isolates. A pairwise alignment of this region from KG37 and KG106 revealed them to be almost identical, so further analyses were carried out only on KG37. As shown in Fig. 4A, this region has a GC content of 30.2%, higher than the 27.4% found in the rest of the KG37 genome. All the proteins contained in this GI were extracted, their amino acid sequences queried against the Virulence Factor Database (VFDB) using BLASTP, and their functions inferred using eggNOG [23]. Five of the twenty-two CDS queried resulted in positive hits against experimentally confirmed virulence factors. Two of these genes are components of a type IV secretory system. Other CDS without hits in the VFD query include three HPs of unknown function, one HP with a non-cytoplasmic domain, and one transmembrane protein containing cytoplasmic, transmembrane, and non-cytoplasmic domains. Other genes found in this GI encode for mobility factors and DNA replication machinery. Since this GI is exclusively present in the highly virulent isolates and contains CDS of proteins orthologous to other VFs, it is a candidate for being classified as a pathogenicity island. The genes contained in this putative pathogenicity island and their descriptions are listed in Supplemental Table 6.

The member proteins of one of the two remaining protein families exclusive to KG37 and KG106 are identical 959-residue HPs of unknown function. Querying their amino acid sequences against the VFDB found a 27% identity with the type IV pilus biogenesis protein PilV of *Yersinia pseudotuberculosis.* A smaller 77-residue HP found downstream from the pilus-associated protein is also only present in the high virulence isolates, but BLASTP searches against the VFDB and the NCBI non-redundant protein sequences database did not return any hits. As shown in Fig. 4B, a structural analysis of these proteins revealed that both contain cytoplasmic, transmembrane, and non-cytoplasmic domains.

Finally, the remaining protein family exclusive to the highly pathogenic isolates is composed of two transposases of different lengths (646 and 171 amino acids) located in non-syntenic regions within their respective genomes. The amino acid sequences of these proteins did not produce any hits when queried against the VFD.

### Putative virulence factors with different copy numbers across isolates

**Fig. 5:**
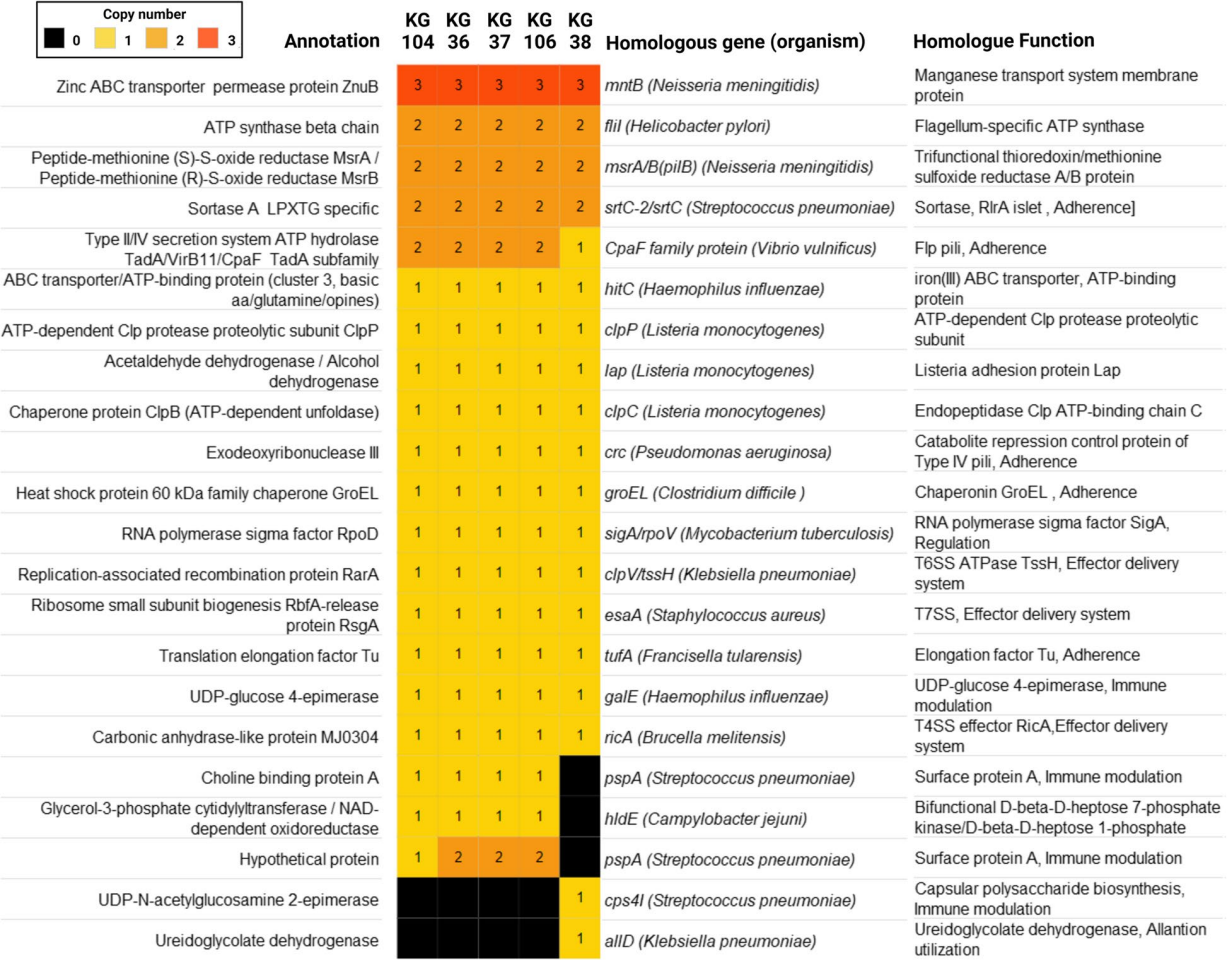
Heat map of *H. ovis* proteins with homologous hits on the VFDB. The color of each cell represents how many paralogous proteins are present in each genome (black = 0, bright yellow = 1, dark yellow = 2, dark orange ≥ 3)

As shown in Fig. 5 and Supplemental Table 7, we identified 22 proteins homologous to experimentally verified virulence factors in other bacterial species. Of these, sixteen are ubiquitously present in all five strains, three are present in all but KG38, two are present only in KG38, and one is present in all strains but with different copy numbers across groups.

**Fig. 6:**
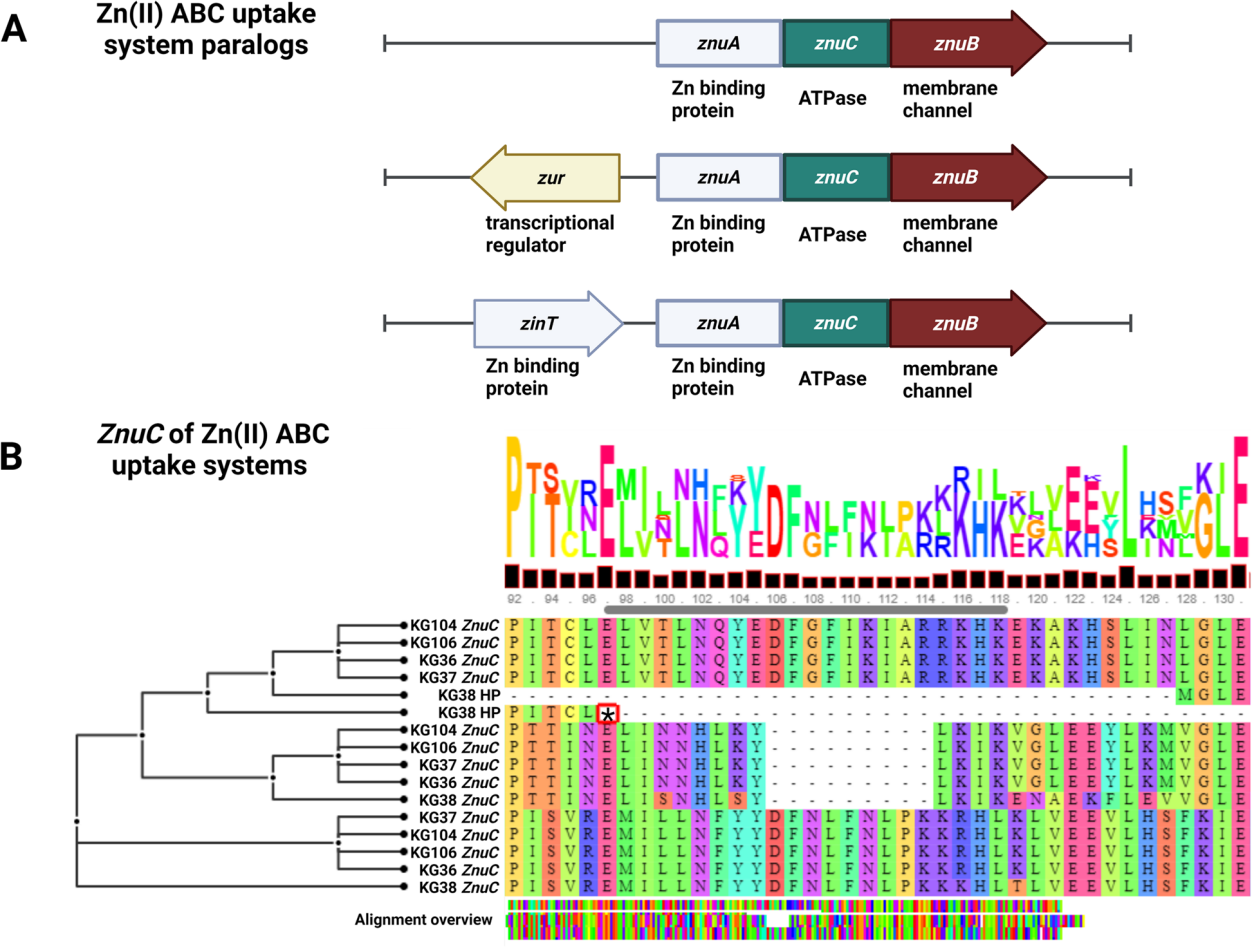
Panel **A** shows the genomic contexts of the three distinct ZnuABC operons across *H. ovis* isolates. CDS colored in red represent proteins that had positive hits in the VFDB. Panel **B** shows amino acids 92 to 130 of the multiple sequence alignment of the ZnuC protein paralogs across *H. ovis* isolates. The black asterisk inside a red box marks a nonsense mutation in one of the KG38 *znuC* CDS

The zinc ABC transporter permease gene *znuB* was identified as a putative virulence factor present in all five isolates, with three paralogous copies in each genome. This gene is part of the ZnuABC zinc transporting system which includes *znuA, znuB, znuC,* and is often accompanied by zinc binding protein *zinT* and zinc uptake regulator *zur* genes [24]. All CDS identified as components of ZnuABC zinc transporting systems are listed in Supplemental Table 8, and an overview of the multiple sequence alignments of ZnuA and ZnuB protein paralogs across genomes is presented in Supplemental Fig. 4. The ZnuABC operon occurs in three different conformations across *H. ovis* isolates, one of which is found downstream from a zinc uptake regulator *zur* CDS and is present in all isolates (Fig. 6). A second operon occurs without any homologs of known ZnuABC associated proteins in its vicinity. While the low virulence strain KG38 has a ZnuABC operon of this type located within LCB 3, the remaining isolates have it situated within LCB 5 (Fig. 2). As shown in Fig. 4, two CDS identified as putative virulence factors in the high virulence strains KG37 and KG106 are found immediately upstream from this ZnuABC operon, raising the possibility that these outer membrane HPs also play a role in Zinc homeostasis. Finally, the third type of ZnuABC operon is downstream from a *zinT* CDS. Low virulence isolate KG38 has a terminal nonsense sequence variation in the *znuc* CDS 286G > T changing a glutamic acid to a stop codon (E97X). This sequence variation leaves the *zinT*-associated ZnuABC operon in KG38 without a functional cytoplasmic ATPase component.

**Fig. 7:**
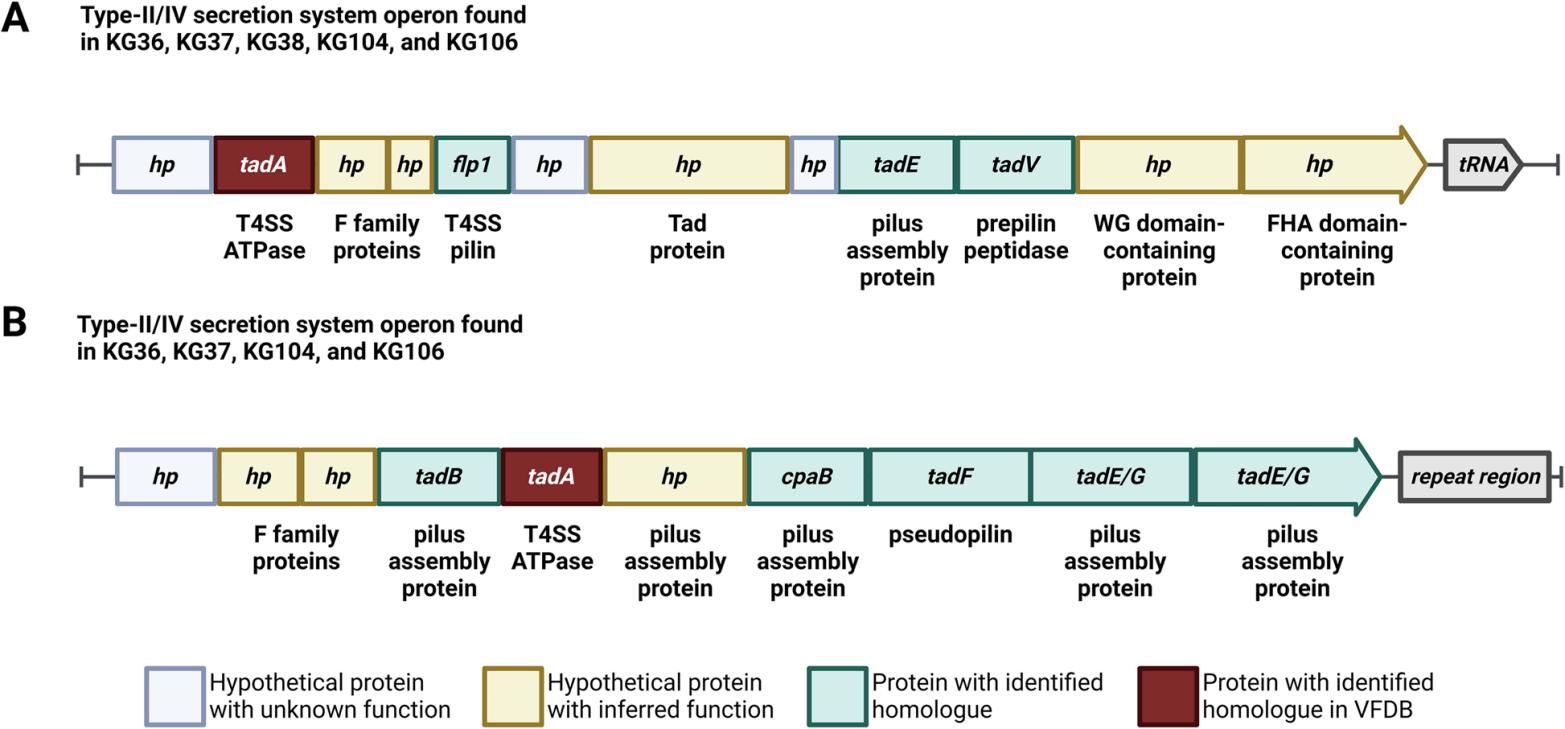
Genomic contexts of Type-II/IV secretion system operons. CDS colored in light blue represent hypothetical proteins of unknown function, green CDS represent hp with inferred function, yellow CDS represent known CDS, and red CDS represent *tadA*

The CDS for *tadA*, a component of T4SS, is found in two paralogous copies across each of the medium and high virulence isolates. The copy of *tadA* ubiquitous to all five isolates is found in an operon with several HPs upstream from a tRNA gene cluster. Three of these HPs are homologous to components of Type II secretion systems including a prepilin peptidase. The second copy of *tadA* is exclusive to the high and medium virulence isolates. This CDS is found within a T4SS operon that includes *tadA*, *tadB*, several HPs, and two CDS for pilus assembly proteins* (flp)*. This conserved operon in the medium and high virulence isolates is entirely absent in KG38. These secretion system-associated operons are shown in Fig. 7.

### Putative virulence factors absent or exclusive to low virulence KG38

As shown in Fig. 5, there are three putative virulence factors present in the high and medium virulence isolates that are absent in KG38. Choline binding protein A is a surface protein made up of a binding module that is anchored to choline on the bacterial cell wall and a functional module that can bind to many host proteins including immunoglobulins. The medium to high virulence strains also has two CDS for hypothetical surface lipoproteins with homologous regions to *Streptococcus pneumoniae* PspA and with *Erysipelothrix rhusiopathiae* Spa; however, KG104 contains only one CDS. Finally, glycerol-3-phosphate cytidylyltransferase CDSs are also present in the middle and high virulence isolates and absent in KG38. This protein mediates the synthesis of cell wall phospholipids. Its homologue found in the VFDB, *hldE*, is a bifunctional protein that is also used in the biosynthesis of lipopolysaccharide in enterotoxigenic *Escherichia coli* [25]*.*

## Discussion

In this study, we assembled the complete genomes of five *H. ovis* isolates from the uterus of dairy cows (*Bos taurus*) and identified organizational genomic structures, a sequence variation, and CDS associated with low and high virulence phenotypes. Previously published *H. ovis* genomes were of draft quality and did not allow for accurate structural analyses [13]. We applied recently developed bioinformatics tools to construct long-read complete genome assemblies that are then improved by short-read polishing methods [20]. The resulting five *H. ovis* assemblies are the first publicly available complete reference-quality genomes for the species. Despite the increasing interest in *H. ovis*, a significant knowledge gap still exists with respect to its genomic diversity, as evidenced by the dearth of publicly available genomes. These newly generated complete genomes will provide a valuable resource for future studies to use short-read-only reference-guided methods for accurate and efficient assemblies.

The phylogenetic tree built using available *H. ovis* 16s rRNA sequences found that the low virulence isolate KG38 forms a clade shared only with the *H. ovis* Tongji strain which constitutes an outgroup from the rest of the *H. ovis* taxon. Since 16s rRNA may not always provide enough certainty to make species and strain level analyses, we built a maximum-likelihood phylogenetic tree using 445 homologous genes in the *Helcococcus* genus which verified some of the 16s rRNA taxonomic findings [26]. Finally, the ANIb pairwise comparisons found evidence that KG38 may represent a novel species within the genus *Helcococcus*. High throughput ANI analyses have identified a valley of genetic discontinuity in pairwise ANI genome comparisons between 83 and 95%. This means most intra-species pairwise comparisons have > 95% ANI, and comparisons of separate species within the same genus have ANI < 83%. Isolate KG38 has mean pairwise ANI with the rest of *H. ovis* isolates of 87%, making it one of the rare 0.2% of sequenced bacterial organisms to fall within the valley of genetic discontinuity [21]. A recent WGS study of 12,000 *Clostridioides difficile* isolates also identified cryptic clades with ANI values within the range of ANI genetic discontinuity [27]. Rare cases like these will provide opportunities to explore the biological foundation of the genetic discontinuity phenomenon. KG38’s closest relative, the *H. ovis* Tongji strain, which originates from a human glass eye infection in China, was found to have an atypical biochemical profile for *H. Ovis* and was also suggested to be a distinct strain or species [12]. Whole genome sequencing of the Tongji strain may be necessary to fully resolve this clade of the *H. ovis* taxon.

Structurally, KG38’s genome is the most dissimilar to all other *H. ovis* genomes, displaying several LCB rearrangements flanked by transposable elements and prophage sequences. One of these rearrangements is likely to have virulence phenotype implications since it is associated with the absence of the collagen adhesin precursor CDS found in all other isolates, leaving KG38 without any copies of this gene. Deletion of the collagen adhesin in *Enterococcus faecalis* caused attenuated virulence in a rat endocarditis model [28]. More than 10% of strain KG38’s genome is made up of gene sequences originating from six different prophage regions. This is an unusually high number of prophage sequences for a 1.8 Mb size genome, which according to recent studies would be expected to contain fewer than three prophages [29]. Finally, ICEs are responsible for the presence of a T4SS-like operon in all five evaluated genomes as well as a second set of T4SS-associated CDS in the genome of the highly virulent KG106. A T2SS-like operon is also present in the medium and high virulence strains but is not associated with ICEs. Bacterial secretion complexes in Gram-positive organisms can fulfill a variety of virulence-associated functions like adherence, horizontal gene transfer, and translocation of virulence factors into the host [30]. These secretion system operons in *H. ovis* contain many HPs and their structure and functions remains to be studied.

The putative pathogenicity island found exclusively in the high virulence strains, KG37 and KG106, is a primary candidate for containing virulence determinants that confers these isolates their relatively high virulence phenotype. There are also two HPs with transmembrane and non-cytoplasmic domains that could account for the elevated virulence. These proteins have no known function and are located upstream from one of the three ZnuABC operons in the high virulence isolates. These zinc transporters are required for virulence in some animal bacterial pathogens as they are used to overcome zinc sequestration by the host [31, 32]. Although all isolates in this study carry three paralogous ZnuABC CDS, each of these operons has slight conformational variations (Fig. 6). One of the operons contains the CDS for ZinT, a membrane protein subordinate to ZnuA, that is strongly induced in *E. coli* adhering to epithelial cells in vitro. ZinT is secreted extracellularly and may induce metal starvation conditions. The nonsense mutation found in the *znuC* gene of the ZinT-associated ZnuABC operon in KG38 raises the possibility this strain may have a lower virulence phenotype from an impaired ability to overcome host zinc sequestration [24].

Although KG38 has many of the VFs identified in this study in common with the rest of the more virulent isolates, it is missing four putative VFs that are present in the high and medium virulence isolates. These CDS are associated with adherence (collagen binding precursor), immune evasion (choline binding protein A and PspA-like HP) and with cell wall synthesis (glycerol-3-phosphate cytidylyltransferase). *H. ovis* can invade various surfaces such as epithelial, endothelial, and synovial tissues across many animal hosts both in the context of single-pathogen or mixed infections. It is likely there are different combinations of VFs required to optimize colonization of each tissue type in each host. Gene expression and disruption studies are necessary to verify the role and function of the putative VFs identified in this study.

## Conclusions

This study presented the first comparative genomic analysis of five complete *H. ovis* genomes and identified putative virulence determinants for the bacterium. Phylogenetic and ANI analyses revealed that the low virulence isolate KG38 forms a distinct clade with the cryptic *H. ovis* Tongji strain, suggesting a potential novel species within the genus *Helcococcus*. A newly described putative pathogenicity island and two transmembrane HPs may account for increased virulence in the species. Additionally, variations in number and conformation of ZnuABC operons, including a nonsense mutation in KG38, raise the possibility of zinc sequestration capacity being a determinant of *H. ovis* virulence. Overall, this study contributes valuable genomic resources for future research in *H. ovis*, sheds light on its genomic diversity and evolutionary relationships, and identifies potential virulence factors, providing a foundation for further investigations into the pathogenesis of this bacterium.

## Methods

### Metritis diagnosis and uterine sample collection

In this study, a total of five lactating Holstein Friesian cows (*Bos taurus)* from the University of Florida’s Dairy Research Unit in north central Florida were used. Each cow had a uterine swab collected and uterine discharge evaluated at four, six, and eight days postpartum. The uterine discharge was scored on a 5-point scale [33]. Score 1 indicates normal lochia, viscous, clear, red, or brown discharge that was not fetid; Score 2 indicates cloudy mucoid discharge with flecks of pus; Score 3 indicates mucopurulent discharge that was not fetid with less than 50% pus; Score 4 indicates mucopurulent discharge that was not fetid with more than 50% pus; and Score 5 indicates fetid red-brownish, watery discharge. Cows with uterine discharge scores of 1–4 were considered healthy, whereas those with a score of 5 were diagnosed with metritis. Uterine discharge samples were collected from healthy cows and from those diagnosed with metritis using a sterile polyester culture swab. The swab contents were then suspended in BHI broth with 30% glycerol and stored at -80 °C.

### Bacteria isolation and virulence group designation

To isolate and select *H. ovis* from uterine discharge samples, 20 µL of the discharge suspension was streaked onto *Helcococcus* selective agar. The agar plates were incubated for 72 h at 36 °C under aerobic conditions with 6% CO2 [2]. Following incubation, individual colonies were selected based on morphology and sub-cultured for further analysis. Identification of the isolates was performed via 16S rRNA gene sequence analysis [13]. Virulence group designation of the *H. ovis* isolates in this study was derived from previous experiments using a *Galleria mellonella* infection model [14].

### Whole genome sequencing

For Illumina short-read sequencing, genomic DNA (gDNA) was extracted using the DNeasy blood and tissue kit following the manufacturer’s instructions (Qiagen). Genomic DNA purity was measured using a NanoDrop 2000 spectrophotometer; final DNA concentration was confirmed with a Qubit 2.0 Fluorometer. Sample libraries were prepared using the Illumina DNA Prep kit and IDT 10 bp UDI indices and sequenced on an Illumina NextSeq 2000, producing 2 × 151 bp reads. Demultiplexing, quality control and adapter trimming were performed with bcl-convert (v3.9.3).

For long-read sequencing, gDNA was extracted using the Monarch HMW DNA Extraction Kit following the protocol for high molecular weight DNA extraction from bacteria (NEB #T3060, New England Biolabs). Genomic DNA purity was measured using a NanoDrop 2000 spectrophotometer; final concentration was confirmed with a Qubit 2.0 Fluorometer (Thermo Scientific). The integrity of the extracted gDNA was evaluated via agarose gel electrophoresis to visually assess that the final product contained a large portion of DNA fragments longer than 20 Kbp and little shearing. Nanopore sequencing samples were prepared using Oxford Nanopore’s “Genomic DNA by Ligation” kit and protocol. All samples were run on Nanopore R9 flow cells (R9.4.1) on a MinION. Post-sequencing, we used Guppy, version 5.0.16 in high-accuracy base calling mode.

### Genome assembly and annotation

De novo assembly of each genome was performed following long read first hybrid assembly methods outlined by Wick et al., 2023. Fastp (v0.23.2) was used with the default settings for quality control of Illumina reads and Filtlong (v0.2.1, https://github.com/rrwick/Filtlong) was used for quality control of ONT reads with a minimum read length of 6 kbp. Filtered ONT reads quality metrics were calculated using NanoPack [34]. Trycycler (v0.5.3) was initially used to assemble the oxford nanopore reads using Flye [35], miniasm/Minipolish [36], and Raven (https://github.com/lbcb-sci/raven) to independently assemble subsampled reads. The resulting assembly was polished with Medaka (v1.7.2, https://github.com/nanoporetech/medaka) using the model settings for MinION R9.4.1 and Guppy version 5.0.16 in high-accuracy base calling mode [37]. The resulting long-read assembly was polished with Polypolish (v0.5.0) short-read polishing, followed by POLCA short-read polishing using the Illumina reads on default settings and with manual curation to look for unexpected clusters of base changes reads with manual curation [38, 39]. The origin of replication (ORI) was identified using ORI-Finder 2022. The chromosome was oriented, and residues were re-numbered to start at the most strongly supported ORI [40]. Assembly quality was assessed using Benchmarking Universal Single-Copy Orthologs (v4.1.2) [41]. Genome annotations were carried out using the genome annotation Service in BV-BRC using the RAST tool kit [42].

### Genome alignment and taxonomy

Multiple genome alignment of all five genomes was performed using progressiveMauve [43]. A separate MAUVE multiple sequence alignment was carried out for the four isolates with pairwise ANIs greater than 95% (KG36, KG104, KG37, and KG106) to identify any smaller-scale structural differences found in the region of LCBs 5, 6, and 7 from the initial alignment. A maximum likelihood phylogenetic tree was constructed on MEGA11 using 16s rRNA sequences extracted from the genomes assembled in this study and with publicly available 16s rRNA sequences of *H. kunzii* and other *H. ovis* isolates. Whole genomes of *H. ovis* and the type strains of other species of the genus *Helcococcus* were used to create a phylogenetic tree using the BV-BRC codon tree pipeline using 500 single-copy PGFams [44]. Phylogenetic trees were visualized and annotated using Interactive Tree of Life (iTOL v5) webtool [45]. Average nucleotide identities (ANI) were calculated based on BLAST + ANIb using the JSpecies web server [46]. ANIb values were used to measure the genetic distance between the two *H. ovis* clades and to contextualize them within the broader *Helcococcus* genus. All sequences used in these phylogenetic analyses are listed in Supplemental Table 9.

### Plasmids and mobile genetic elements

Plassembler (v0.1.5) was used to screen the filtered Illumina short reads for any plasmids that may have been missed during the genome assembly process (https://github.com/gbouras13/plassembler). The Phaster web service was used to identify prophage regions within the genomes. The annotated genomes were uploaded to the Phaster website, and the service was run using default parameters. ICEs were identified by using the ICEfinder webtool of ICEberg v.2.0 [47].

### Identification of putative virulence factors

To identify patterns that may be associated with the medium and low virulence isolates, the VFDB was searched using BLASTP against the complete proteome of each *H. ovis* genome [48, 49]. BLASTP hits with a minimum value of 30% identity, 70% coverage, and minimum bit scores of 50 were retained for further analysis. Furthermore, the comparative systems service on the BV-BRC website was used to identify and compare proteins across the selected bacterial genomes. This service identifies orthologous proteins and assigns them to protein families. Using the BV-BRC Protein Family Sorter tool allowed us to visualize the distribution of protein families across *H. ovis* genomes. The global (PGFam) method was used to identify and classify protein families within the selected bacterial genomes. The PGFam method is a homology-based approach that uses hidden Markov models (HMMs) to identify protein families [44]. Protein families were then used to generate gene family presence and absence comparative diagrams between strains. Protein families exclusive to the low, medium, or high virulence groups were identified and a less conservative BLASTP homology search on the Virulence Factor Database was performed. Proteins with negative E values and with bit scores greater than 50 were retained [50]. EggNOG v5.0 was used to identify orthologs and infer the function of proteins of interests [23]. The InterProScan software was also used to predict protein function and to classify protein domains [51].

### Supplementary Information

**Additional file 1: Supplemental Figure 1.** Multiple sequence alignments of high and medium virulence *H. ovis* genomes using MAUVE.

**Additional file 2: Supplemental Figure 2.** Putative prophage sequences identified and annotated using the PHASTER web server.

**Additional file 3: Supplemental Figure 3.** Genomic context of incomplete conserved putative prophage region (Region 1) encoding for two subunits of a heterodimeric efflux transporter protein.

**Additional file 4: Supplemental Figure 4.** First 70 amino acids of the multiple sequence alignments and alignment overview boxes of ZnuA and ZnuB protein paralogs across all five *H.ovis* genomes.

**Additional file 5: Supplemental Table 1.** Clinical and phenotypic information of *Helcococcus ovis* isolates.

**Additional file 6: Supplemental Table 2. **Descriptive statistics for Illumina and ONT filtered reads used to build hybrid genome assemblies.

**Additional file 7: Supplemental Table 3. **ANIb comparison between all available *Helcococcus ovis* genomes and the type strain for each species of the *Helcococcus *genus.

**Additional file 8: Supplemental Table 4. **List of prophage regions and their features flagged by PHASTER within *Helcococcus ovis* genomes.

**Additional file 9: Supplemental Table 5. **List of integrative and conjugative elements and their features found within *Helcococcus ovis* genomes.

**Additional file 10: Supplemental Table 6. **List of CDS found in pathogenicity island of high virulence *Helcococcus ovis *strains.

**Additional file 11: Supplemental Table 7. **List of *Helcococcus ovis* CDS with positive hits against experimentally verified virulence factors on the Virulence Factor Database.

**Additional file 12: Supplemental Table 8. **List of features of ZnuC orthologs found in *Helcococcus ovis *genomes. These components of Zinc ABC transporters were used for protein multiple sequence alignment analyses.

**Additional file 13: Supplemental Table 9. **Accession numbers for nucleotide sequences used in Figure 1 phylogenetic analyses.

## Data Availability

The whole-genome sequences and the trimmed reads have been uploaded into the NCBI Sequence Read Archive and can be found under BioProject number PRJNA514352. GenBank accession numbers for the genome assemblies are SAMN10734405 (KG36), SAMN10734406 (KG37), SAMN33847359 (KG38), SAMN33714545 (KG104), and SAMN33714546 (KG106).

## Abbreviations

ANI: Average nucleotide identity
CDS: Coding sequence
gDNA: Genomic DNA
GI: Genomic island
*H. ovis*: *Helcococcus ovis*
HP: Hypothetical protein
*H. kunzii*: *Helcococcus kunzii*
ICE: Integrative and conjugative element
LCB: Locally collinear block
*M. bovis*: *Mycoplasma bovis*
ONT: Oxford Nanopore Technologies
ORI: Origin of replication
T4SS: Type IV secretion system
*T. pyogenes*: *Trueperella pyogenes*
VF: Virulence factor
VFDB: Virulence Factor Database
