# Supplementary figures and images for: Sequencing and characterization of Helcococcus ovis: a comprehensive comparative genomic analysis of virulence
Source: BMC Genomics. 2023 Aug 30;24:501. doi: 10.1186/s12864-023-09581-1 (PMC10466703; doi:10.1186/s12864-023-09581-1)

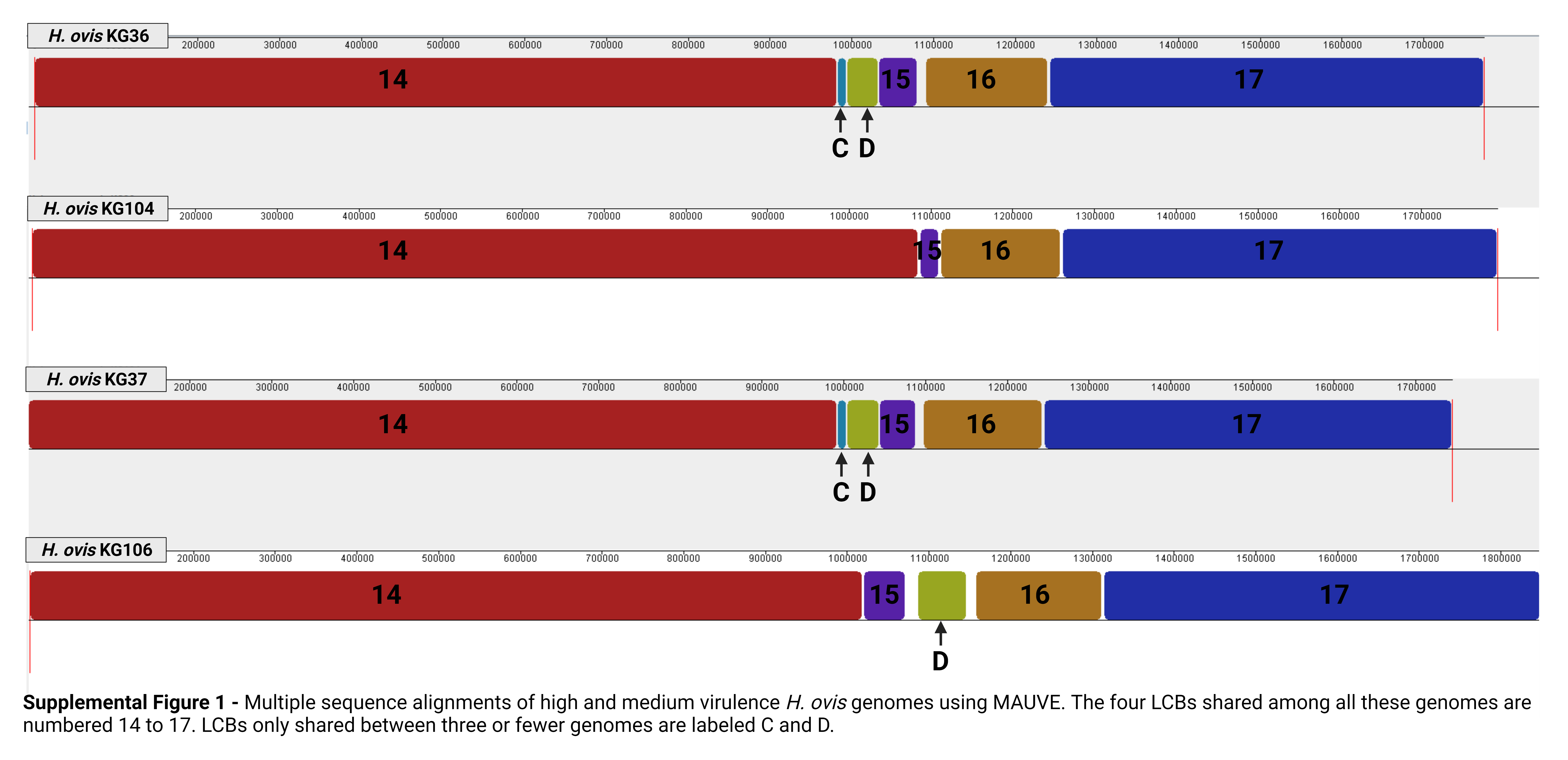

Supplement: Supplementary file 1 — Additional file 1: Supplemental Figure 1. Multiple sequence alignments of high and medium virulence H. ovis genomes using MAUVE. [file 12864_2023_9581_MOESM1_ESM.png]

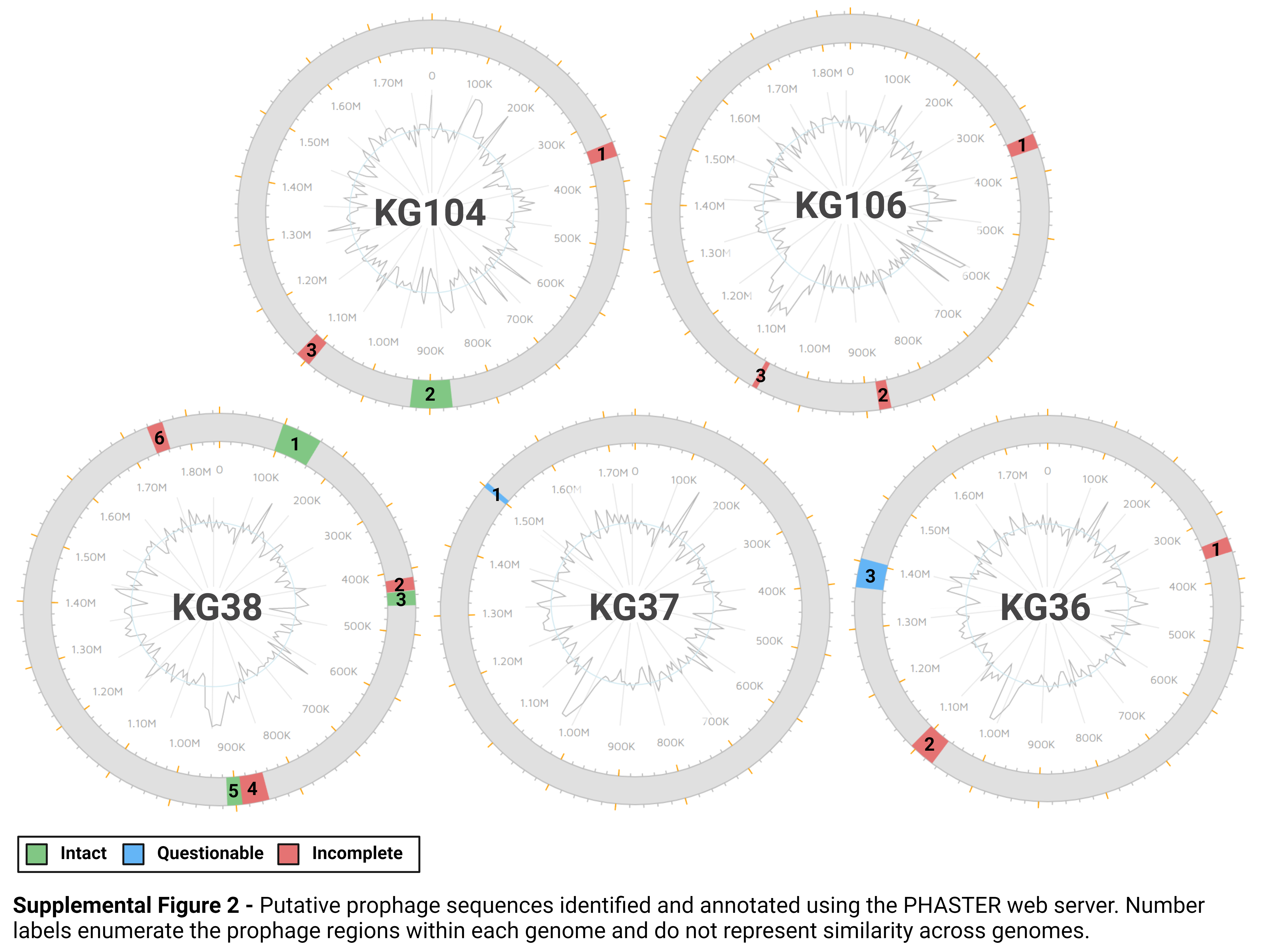

Supplement: Supplementary file 2 — Additional file 2: Supplemental Figure 2. Putative prophage sequences identified and annotated using the PHASTER web server. [file 12864_2023_9581_MOESM2_ESM.png]

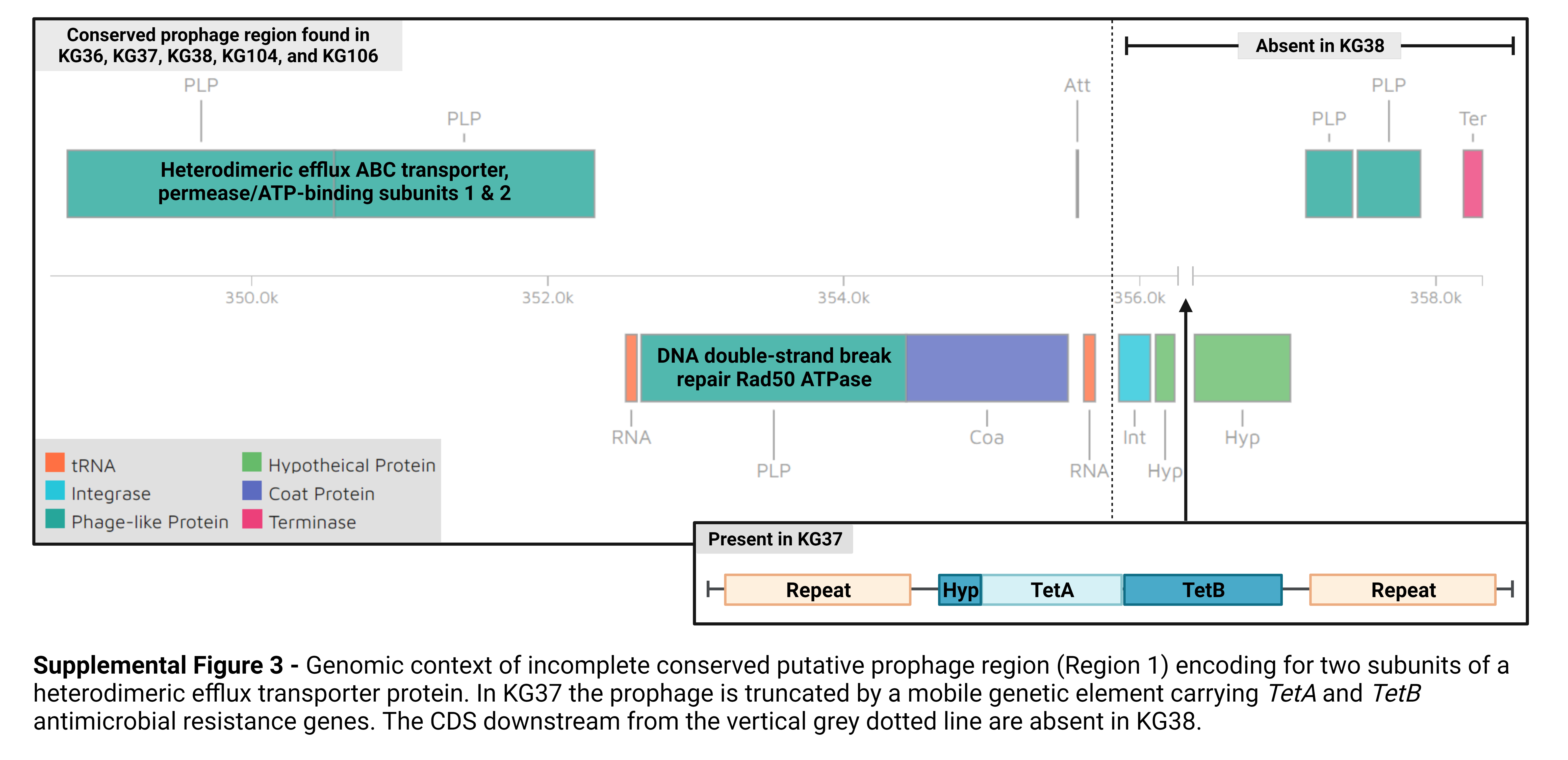

Supplement: Supplementary file 3 — Additional file 3: Supplemental Figure 3. Genomic context of incomplete conserved putative prophage region (Region 1) encoding for two subunits of a heterodimeric efflux transporter protein. [file 12864_2023_9581_MOESM3_ESM.png]

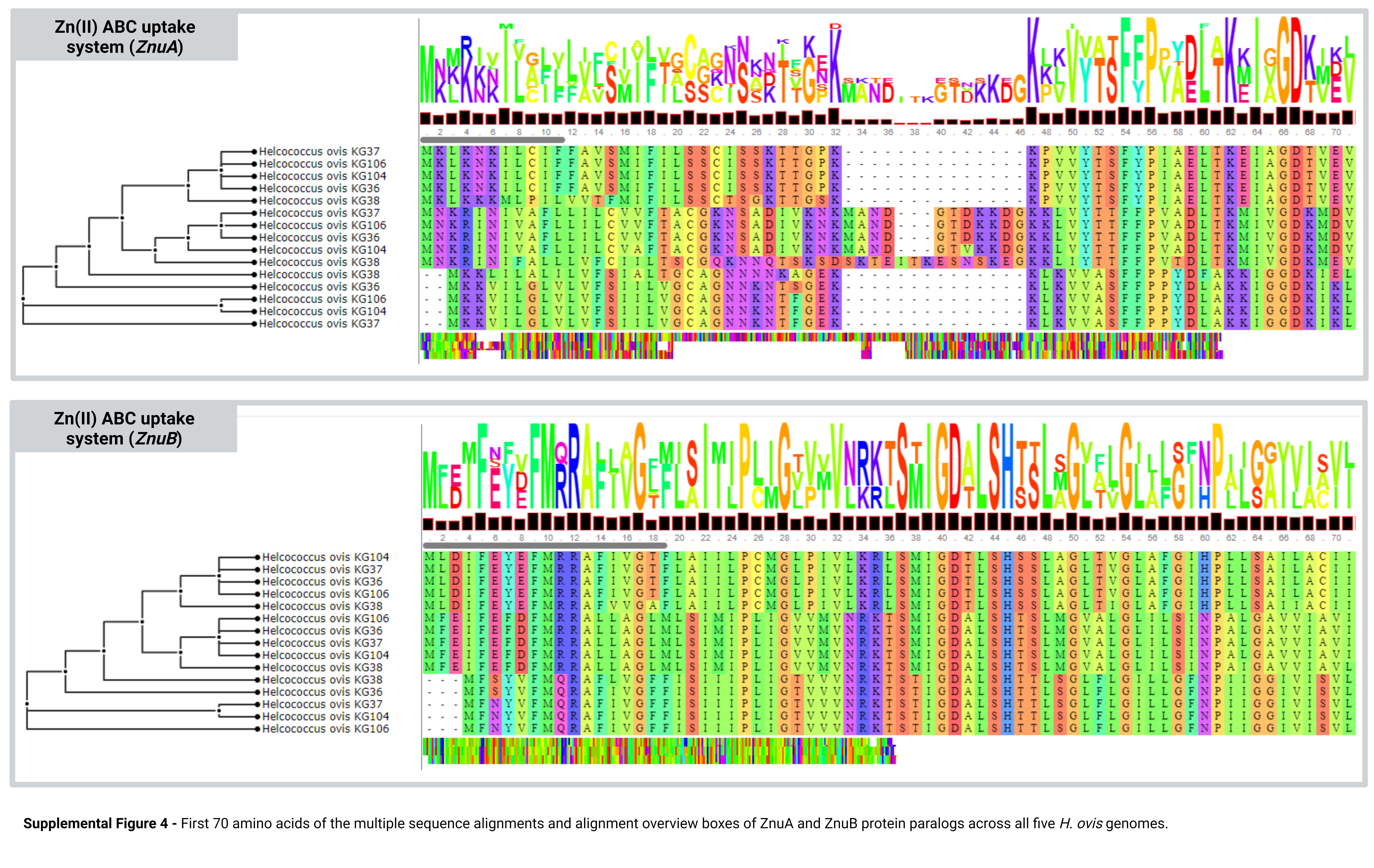

Supplement: Supplementary file 4 — Additional file 4: Supplemental Figure 4. First 70 amino acids of the multiple sequence alignments and alignment overview boxes of ZnuA and ZnuB protein paralogs across all five H.ovis genomes. [file 12864_2023_9581_MOESM4_ESM.png]
