# Supplementary material for: Sequencing and characterization of Helcococcus ovis: a comprehensive comparative genomic analysis of virulence
Source: BMC Genomics. 2023 Aug 30;24:501. doi: 10.1186/s12864-023-09581-1 (PMC10466703; doi:10.1186/s12864-023-09581-1)
Supplement: Supplementary file 5 — Additional file 5: Supplemental Table 1. Clinical and phenotypic information of Helcococcus ovis isolates. [file 12864_2023_9581_MOESM5_ESM.docx]

**Supplemental Table 1 -** Clinical and phenotypic information of *Helcococcus ovis* isolates.

| **Strain No.** | **Species** | **Isolation date** | **Isolation location** | **Health Status** | **Virulence** | **Reference** |
| --- | --- | --- | --- | --- | --- | --- |
| KG36 | *Helcococcus ovis* | 12/2/2015 | North central Florida, USA | Metritis | Medium | Cunha et al,. 2018 |
| KG37 | *Helcococcus ovis* | 12/2/2015 | North central Florida, USA | Metritis | High | Cunha et al,. 2018 |
| KG38 | *Helcococcus ovis* | 12/2/2015 | North central Florida, USA | Healthy | Low | Cunha et al,. 2023 |
| KG104 | *Helcococcus ovis* | 9/5/2020 | North central Florida, USA | Metritis | Medium | Cunha et al,. 2023 |
| KG106 | *Helcococcus ovis* | 9/5/2020 | North central Florida, USA | Metritis | High | Cunha et al,. 2023 |
