# Supplementary material for: Sequencing and characterization of Helcococcus ovis: a comprehensive comparative genomic analysis of virulence
Source: BMC Genomics. 2023 Aug 30;24:501. doi: 10.1186/s12864-023-09581-1 (PMC10466703; doi:10.1186/s12864-023-09581-1)
Supplement: Supplementary file 6 — Additional file 6: Supplemental Table 2. Descriptive statistics for Illumina and ONT filtered reads used to build hybrid genome assemblies. [file 12864_2023_9581_MOESM6_ESM.docx]

**Supplemental Table 2 -** Descriptive statistics for Illumina and ONT filtered reads used to build hybrid genome assemblies.

| **Ilumina Reads** | | | | | | | |  | | | | | | | | | |  | | | | |
| --- | --- | --- | --- | --- | --- | --- | --- | --- | --- | --- | --- | --- | --- | --- | --- | --- | --- | --- | --- | --- | --- | --- |
| **Sample Name** | | | **Total Reads** | | | **Total bp** | | | **Mean read length** | | | **Total bp > Q30** | | | | | **% bp > Q30** | | **Coverage** | |  |  |
| KG36 | | | 6556292 | | | 957282596 | | | 146 | | | 879193287 | | | | | 96.53 | | 531x | |  |  |
| KG37 | | | 6981392 | | | 1033712000 | | | 148 | | | 955797686 | | | | | 92.46 | | 574x | |  |  |
| KG38 | | | 7028706 | | | 1018224000 | | | 145 | | | 937148079 | | | | | 92.03 | | 565x | |  |  |
| KG104 | | | 21339794 | | | 2723238000 | | | 127 | | | 2540915000 | | | | | 93.3 | | 1512x | |  |  |
| KG106 | | | 22389138 | | | 2822403000 | | | 126 | | | 2636350000 | | | | | 93.41 | | 1568x | |  |  |
|  |  | | |  | | |  | | |  | | | |  | |  |  |  |  |  |  |  |
| **ONT Reads** | | | | | | | | | | | | |  | | | | | | | | | |
| **Sample Name** | | **Total Reads** | | | **Total bp** | | | **Mean read length** | | | **Read length N50** | | | | **Mean read quality** | | | **Total bp > Q12** | | **% bp > Q12** | | **Coverage** |
| KG36 | | 10069 | | | 98041332 | | | 9737 | | | 9732 | | | | 12.4 | | | 56000000 | | 59.4 | | 54x |
| KG37 | | 12445 | | | 133289557 | | | 10710 | | | 10995 | | | | 12.4 | | | 77000000 | | 59.9 | | 74x |
| KG38 | | 16235 | | | 127190531 | | | 7834 | | | 8079 | | | | 12.3 | | | 71000000 | | 58.1 | | 71x |
| KG104 | | 17684 | | | 139975773 | | | 7915 | | | 8302 | | | | 12.3 | | | 764000000 | | 57.1 | | 78x |
| KG106 | | 40993 | | | 370057573 | | | 6663 | | | 9963 | | | | 12.8 | | | 234000000 | | 66.9 | | 205x |
