# Supplementary material for: Sequencing and characterization of Helcococcus ovis: a comprehensive comparative genomic analysis of virulence
Source: BMC Genomics. 2023 Aug 30;24:501. doi: 10.1186/s12864-023-09581-1 (PMC10466703; doi:10.1186/s12864-023-09581-1)
Supplement: Supplementary file 7 — Additional file 7: Supplemental Table 3. ANIb comparison between all available Helcococcus ovis genomes and the type strain for each species of the Helcococcus genus. [file 12864_2023_9581_MOESM7_ESM.docx]

**Supplemental Table 3 -** ANIb comparison between all available *Helcococcus ovis* genomes and the type strain for each species of the *Helcococcus* genus.

| **ANIb results** |  |  |  |  |  |  |  |  |  |  |
| --- | --- | --- | --- | --- | --- | --- | --- | --- | --- | --- |
|  | **Helcococus ovis KG38** | **Helcococus ovis KG36** | **Helcococus ovis KG37** | **Helcococus ovis KG104** | **Helcococus ovis KG106** | **Helcococcus kunzii ATCC 51366 [T]** | **Helcococcus sueciensis DSM 17243 [T]** | **Helcococcus massiliensis Marseille-P4590 [T]** | **Helcococus ovis KG39.fna** | **Helcococus ovis KG40.fna** |
| **Helcococus ovis KG38** | * | 87.66 | 87.72 | 86.51 | 87.75 | 74.11 | 73.68 | 72.07 | 87.68 | 87.64 |
| **Helcococus ovis KG36** | 87.88 | * | 98.98 | 98.41 | 98.60 | 74.26 | 73.35 | 71.14 | 98.96 | 99.99 |
| **Helcococus ovis KG37** | 87.93 | 98.75 | * | 98.25 | 98.60 | 74.12 | 73.06 | 71.01 | 99.80 | 98.75 |
| **Helcococus ovis KG104** | 87.38 | 98.40 | 98.49 | * | 98.28 | 74.00 | 73.07 | 71.07 | 98.47 | 98.40 |
| **Helcococus ovis KG106** | 87.99 | 98.23 | 98.40 | 97.93 | * | 74.17 | 72.91 | 71.96 | 98.37 | 98.24 |
| **Helcococcus kunzii ATCC 51366 [T]** | 73.69 | 73.52 | 73.39 | 73.47 | 73.30 | * | 72.80 | 70.76 | 73.43 | 73.50 |
| **Helcococcus sueciensis DSM 17243 [T]** | 72.93 | 72.72 | 72.66 | 72.67 | 72.49 | 72.81 | * | 73.28 | 72.68 | 72.72 |
| **Helcococcus massiliensis Marseille-P4590 [T]** | 71.18 | 70.31 | 70.29 | 70.51 | 71.23 | 70.64 | 72.85 | * | 70.31 | 70.35 |
| **Helcococus ovis KG39.fna** | 87.68 | 98.65 | 99.55 | 97.93 | 98.20 | 74.21 | 73.13 | 71.16 | * | 98.62 |
| **Helcococus ovis KG40.fna** | 87.68 | 99.95 | 98.90 | 98.22 | 98.47 | 74.18 | 73.28 | 71.20 | 98.92 | * |
