# Supplementary material for: Sequencing and characterization of Helcococcus ovis: a comprehensive comparative genomic analysis of virulence
Source: BMC Genomics. 2023 Aug 30;24:501. doi: 10.1186/s12864-023-09581-1 (PMC10466703; doi:10.1186/s12864-023-09581-1)
Supplement: Supplementary file 8 — Additional file 8: Supplemental Table 4. List of prophage regions and their features flagged by PHASTER within Helcococcus ovis genomes. [file 12864_2023_9581_MOESM8_ESM.docx]

**Supplemental Table 4 -** List of prophage regions and their features flagged by PHASTER within *Helcococcus ovis* genomes.

| Strain | Region | Region Length | Completeness | Score | CDS | Region Position | Most Common Phage | GC % |
| --- | --- | --- | --- | --- | --- | --- | --- | --- |
| KG104 | 1 | 22.4Kb | incomplete | 40 | 9 | 338954-361361 | PHAGE_Lactoc_bIL286_NC_002667(1) | 0.2486 |
| KG104 | 2 | 63.2Kb | intact | 120 | 62 | 866337-929622 | PHAGE_Geobac_E2_NC_009552(6) | 0.3478 |
| KG104 | 3 | 23.1Kb | incomplete | 30 | 13 | 1092626-1115758 | PHAGE_Clostr_phi3626_NC_003524(2) | 0.2966 |
| KG106 | 1 | 22.4Kb | incomplete | 40 | 9 | 341332-363738 | PHAGE_Lister_LMTA_34_NC_042048(1) | 0.2481 |
| KG106 | 2 | 18.1Kb | incomplete | 40 | 10 | 862964-881155 | PHAGE_Sulfol_SMV1_NC_023585(1) | 0.2545 |
| KG106 | 3 | 8.1Kb | incomplete | 30 | 9 | 1068644-1076804 | PHAGE_Lister_B054_NC_009813(2) | 0.3682 |
| KG36 | 1 | 22.4Kb | incomplete | 40 | 10 | 335909-358315 | PHAGE_Plankt_PaV_LD_NC_016564(1) | 0.2482 |
| KG36 | 2 | 39.6Kb | incomplete | 50 | 21 | 1067596-1107272 | PHAGE_Clostr_phi3626_NC_003524(2) | 0.2931 |
| KG36 | 3 | 43.2Kb | questionable | 70 | 45 | 1361438-1404658 | PHAGE_Faecal_FP_Lugh_NC_047912(7) | 0.2958 |
| KG37 | 1 | 8.1Kb | questionable | 70 | 12 | 1498139-1506268 | PHAGE_Entero_fiAA91_ss_NC_022750(2) | 0.2732 |
| KG38 | 1 | 61.6Kb | intact | 100 | 45 | [97521-159154](https://phaster.ca/submissions/ZZ_9cf8f19fb0#region_dna0) | PHAGE_Coryne_Lederberg_NC_048790(6) | 0.3304 |
| KG38 | 2 | 19.7Kb | incomplete | 30 | 28 | [413062-432824](https://phaster.ca/submissions/ZZ_9cf8f19fb0#region_dna1) | PHAGE_Paenib_Vegas_NC_028767(6) | 0.2877 |
| KG38 | 3 | 22.3Kb | intact | 110 | 30 | [433616-455952](https://phaster.ca/submissions/ZZ_9cf8f19fb0#region_dna2) | PHAGE_Geobac_GBSV1_NC_008376(10) | 0.3024 |
| KG38 | 4 | 46.6Kb | incomplete | 30 | 21 | [849373-896015](https://phaster.ca/submissions/ZZ_9cf8f19fb0#region_dna3) | PHAGE_Coryne_Lederberg_NC_048790(5) | 0.3046 |
| KG38 | 5 | 23Kb | intact | 120 | 27 | [890240-913317](https://phaster.ca/submissions/ZZ_9cf8f19fb0#region_dna4) | PHAGE_Geobac_GBSV1_NC_008376(4) | 0.2928 |
| KG38 | 6 | 24.4Kb | incomplete | 10 | 13 | [1738235-1762723](https://phaster.ca/submissions/ZZ_9cf8f19fb0#region_dna5) | PHAGE_Coryne_Stiles_NC_048789(4) | 0.2904 |
