# Supplementary material for: Sequencing and characterization of Helcococcus ovis: a comprehensive comparative genomic analysis of virulence
Source: BMC Genomics. 2023 Aug 30;24:501. doi: 10.1186/s12864-023-09581-1 (PMC10466703; doi:10.1186/s12864-023-09581-1)
Supplement: Supplementary file 9 — Additional file 9: Supplemental Table 5. List of integrative and conjugative elements and their features found within Helcococcus ovis genomes. [file 12864_2023_9581_MOESM9_ESM.docx]

**Supplemental Table 5 -** List of integrative and conjugative elements and their features found within *Helcococcus ovis* genomes.

| Strain | Name | Location | Length/bp | Type | Prophage |
| --- | --- | --- | --- | --- | --- |
| KG36 | [Region1](https://bioinfo-mml.sjtu.edu.cn/ICEfinder/feature_page_v2.php?job_id=pM1TNlHSl\|ICEfinder_1_0\|Region1) | 219627..225350 | 5724 | Putative IME | N |
| KG36 | [Region2](https://bioinfo-mml.sjtu.edu.cn/ICEfinder/feature_page_v2.php?job_id=pM1TNlHSl\|ICEfinder_4_0\|Region2) | 973410..1032681 | 59272 | Putative ICE with T4SS | N |
| KG36 | [Region3](https://bioinfo-mml.sjtu.edu.cn/ICEfinder/feature_page_v2.php?job_id=pM1TNlHSl\|ICEfinder_5_0\|Region3) | 1060633..1107804 | 47172 | Putative IME | N |
| KG36 | [Region4](https://bioinfo-mml.sjtu.edu.cn/ICEfinder/feature_page_v2.php?job_id=pM1TNlHSl\|ICEfinder_7_0\|Region4) | 1584081..1595662 | 11582 | Putative IME | Y |
| KG37 | [Region1](https://bioinfo-mml.sjtu.edu.cn/ICEfinder/feature_page_v2.php?job_id=pTeRLcnBF\|ICEfinder_4_0\|Region1) | 983691..1041578 | 57888 | Putative ICE with T4SS | N |
| KG38 | [Region1](https://bioinfo-mml.sjtu.edu.cn/ICEfinder/feature_page_v2.php?job_id=pykAVRW1O\|ICEfinder_3_1\|Region1) | 236142..243275 | 7134 | Putative IME without identified DR | N |
| KG38 | [Region2](https://bioinfo-mml.sjtu.edu.cn/ICEfinder/feature_page_v2.php?job_id=pykAVRW1O\|ICEfinder_9_0\|Region2) | 740807..797755 | 56949 | Putative ICE with T4SS | N |
| KG104 | [Region1](https://bioinfo-mml.sjtu.edu.cn/ICEfinder/feature_page_v2.php?job_id=pgHbf8jdv\|ICEfinder_4_0\|Region1) | 826378..973706 | 147329 | Putative ICE with T4SS | N |
| KG104 | [Region2](https://bioinfo-mml.sjtu.edu.cn/ICEfinder/feature_page_v2.php?job_id=pgHbf8jdv\|ICEfinder_5_0\|Region2) | 1093933..1118958 | 25026 | Putative IME | Y |
| KG106 | [Region1](https://bioinfo-mml.sjtu.edu.cn/ICEfinder/feature_page_v2.php?job_id=pklVksQBM\|ICEfinder_1_0\|Region1) | 575806..635574 | 59769 | Putative ICE with T4SS | N |
| KG106 | [Region2](https://bioinfo-mml.sjtu.edu.cn/ICEfinder/feature_page_v2.php?job_id=pklVksQBM\|ICEfinder_4_0\|Region2) | 1026821..1107091 | 80271 | Putative ICE with T4SS | Y |
