# Supplementary material for: Sequencing and characterization of Helcococcus ovis: a comprehensive comparative genomic analysis of virulence
Source: BMC Genomics. 2023 Aug 30;24:501. doi: 10.1186/s12864-023-09581-1 (PMC10466703; doi:10.1186/s12864-023-09581-1)
Supplement: Supplementary file 10 — Additional file 10: Supplemental Table 6. List of CDS found in pathogenicity island of high virulence Helcococcus ovis strains. [file 12864_2023_9581_MOESM10_ESM.docx]

**Supplemental Table 6 -** List of CDS found in pathogenicity island of high virulence *Helcococcus ovis* strains.

| **Product** | **Gene Symbol** | **Length (AA)** | **Homologous gene description** |
| --- | --- | --- | --- |
| Direct repeat (457 bp) | dr | N/A | N/A |
| Transposase | tnp | 81 | IS256 family transposase |
| Hypothetical Protein * | hp | 234 | KilA-N domain-containing protein |
| Hypothetical Protein | hp | 93 | vCD1845 family protein |
| Dna replication helicase loader DnaC/DnaI * | dnaA | 254 | Replication initiator protein A |
| Hypothetical Protein | hp | 49 | ATP-binding protein |
| DNA replication helicase loader DnaC/DnaI | dnaC/I | 159 | transfers helicase to replication origins |
| Hypothetical Protein * | hp | 161 | PcfB family protein (the type iv (conjugal dna-protein transfer or virb) secretory pathway (ivsp) family) |
| TrsK-like protein * | trsK | 189 | type IV secretory system conjugative DNA transfer family protein |
| Site-specific recombinase | ssr | 209 | TnpX site-specific recombinase |
| Site-specific recombinase | ssr | 150 | TnpX site-specific recombinase |
| Transcriptional regulator, Xre family | tr | 32 | Xenobiotic Response Element family transcriptional regulator |
| Hypothetical Protein | hp | 37 | HP of unknown function |
| Hypothetical Protein | hp | 81 | HP with signal peptide and non cytoplasmic domain |
| Hypothetical Protein | hp | 155 | HP of unknown function |
| Conserved membrane spanning protein with endonuclease/ exonuclease/ phosphatase domain | tp | 339 | Transmembrane protein with cytoplasmic, transmembrane, and non-cytoplasmic domains. |
| FIG00532040: hypothetical protein | hp | 63 | Sigma factor |
| Transcriptional regulator, Xre family * | tr | 69 | transcriptional regulation |
| Hypothetical Protein | hp | 130 | HP of unknown function |
| Transcriptional regulator, MerR family | tr | 282 | Transcriptional regulator, MerR family |
| FIG00648277: hypothetical protein | hp | 65 | excisionase |
| Integrase | int | 44 | tyrosine-type recombinase/integrase |
| Integrase | int | 229 | tyrosine-type recombinase/integrase |
| Direct repeat (457 bp) | dr | N/A | N/A |
