# Supplementary material for: Sequencing and characterization of Helcococcus ovis: a comprehensive comparative genomic analysis of virulence
Source: BMC Genomics. 2023 Aug 30;24:501. doi: 10.1186/s12864-023-09581-1 (PMC10466703; doi:10.1186/s12864-023-09581-1)
Supplement: Supplementary file 11 — Additional file 11: Supplemental Table 7. List of Helcococcus ovis CDS with positive hits against experimentally verified virulence factors on the Virulence Factor Database. [file 12864_2023_9581_MOESM11_ESM.docx]

**Supplemental Table 7 –** List of *Helcococcus ovis* CDS with positive hits against experimentally verified virulence factors on the Virulence Factor Database.

| **Protein** | **ID** | **% ID** | **Positives** | **% Coverage** | **Hit** |
| --- | --- | --- | --- | --- | --- |
| fig\|72026.48.peg.1016\| ATP synthase beta chain (EC 3.6.3.14) [Helcococcus ovis KG36 \| 72026.48] | 17/36 | 47 | 27/36 | 75 | (yscN) type III secretion system ATPase YscN [TTSS - Effector delivery system] [Yersinia pestis CO92] Length = 439 Score = 146 bits (368), Expect = 4e-36 Identities = 115/365 (31%), Positives = 179/365 (49%), Gaps = 17/365 (4%) |
| fig\|72026.48.peg.1133\| Type II/IV secretion system ATP hydrolase TadA/VirB11/CpaF, TadA subfamily [Helcococcus ovis KG36 \| 72026.48] | 199/400 | 49 | 281/400 | 70 | (VV1_RS15610) CpaF family protein [Flp pili - Adherence] [Vibrio vulnificus CMCP6] Length = 421 Score = 387 bits (993), Expect = e-108 Identities = 199/400 (49%), Positives = 281/400 (70%), Gaps = 3/400 (0%) |
| fig\|72026.48.peg.116\| Chaperone protein ClpB (ATP-dependent unfoldase) [Helcococcus ovis KG36 \| 72026.48] | 137/248 | 55 | 182/248 | 73 | (clpE) ATP-dependent protease [ClpE - Stress survival] [Listeria monocytogenes EGD-e] Length = 724 Score = 268 bits (686), Expect = 1e-72 Identities = 137/248 (55%), Positives = 182/248 (73%), Gaps = 2/248 (0%) |
| fig\|72026.48.peg.1364\| Heat shock protein 60 kDa family chaperone GroEL [Helcococcus ovis KG36 \| 72026.48] | 335/538 | 62 | 429/538 | 79 | (groEL) chaperonin GroEL [GroEL - Adherence] [Clostridium difficile 630] Length = 542 Score = 648 bits (1672), Expect = 0.0 Identities = 335/538 (62%), Positives = 429/538 (79%), Gaps = 2/538 (0%) |
| fig\|72026.48.peg.1392\| RNA polymerase sigma factor RpoD [Helcococcus ovis KG36 \| 72026.48] | 153/243 | 62 | 199/243 | 81 | (sigA/rpoV) RNA polymerase sigma factor SigA [SigA - Regulation] [Mycobacterium tuberculosis H37Rv] Length = 528 Score = 317 bits (812), Expect = 2e-87 Identities = 153/243 (62%), Positives = 199/243 (81%) |
| fig\|72026.48.peg.1411\| Acetaldehyde dehydrogenase (EC 1.2.1.10) / Alcohol dehydrogenase (EC 1.1.1.1) [Helcococcus ovis KG36 \| 72026.48] | 538/863 | 62 | 676/863 | 78 | (lap) Listeria adhesion protein Lap [Lap - Adherence] [Listeria monocytogenes EGD-e] Length = 866 Score = 1093 bits (2828), Expect = 0.0 Identities = 538/863 (62%), Positives = 676/863 (78%), Gaps = 2/863 (0%) |
| fig\|72026.48.peg.1474\| ABC transporter, ATP-binding protein (cluster 3, basic aa/glutamine/opines) [Helcococcus ovis KG36 \| 72026.48] | 115/221 | 52 | 156/221 | 70 | (bauE) ferric siderophore ABC transporter, ATP-binding protein BauE [Acinetobactin - Nutritional/Metabolic factor] [Acinetobacter baumannii ACICU] Length = 256 Score = 94.0 bits (232), Expect = 1e-20 Identities = 64/221 (28%), Positives = 115/221 (52%), Gaps = 14/221 (6%) |
| fig\|72026.48.peg.1532\| Sortase A, LPXTG specific [Helcococcus ovis KG36 \| 72026.48] | 138/232 | 59 | 188/232 | 81 | (srtC-2/srtC) sortase [RlrA islet - Adherence] [Streptococcus pneumoniae TIGR4] Length = 297 Score = 303 bits (776), Expect = 1e-83 Identities = 138/232 (59%), Positives = 188/232 (81%) |
| fig\|72026.48.peg.1533\| Sortase A, LPXTG specific [Helcococcus ovis KG36 \| 72026.48] | 158/219 | 72 | 186/219 | 84 | (srtC-1/srtB) sortase [RlrA islet - Adherence] [Streptococcus pneumoniae TIGR4] Length = 300 Score = 332 bits (851), Expect = 3e-92 Identities = 158/219 (72%), Positives = 186/219 (84%) |
| fig\|72026.48.peg.1563\| Peptide-methionine (S)-S-oxide reductase MsrA (EC 1.8.4.11) / Peptide-methionine (R)-S-oxide reductase MsrB (EC 1.8.4.12) [Helcococcus ovis KG36 \| 72026.48] | 198/324 | 61 | 236/324 | 72 | (msrA/B(pilB)) trifunctional thioredoxin/methionine sulfoxide reductase A/B protein [MsrAB - Stress survival] [Neisseria meningitidis MC58] Length = 522 Score = 406 bits (1043), Expect = e-114 Identities = 198/324 (61%), Positives = 236/324 (72%), Gaps = 12/324 (3%) |
| fig\|72026.48.peg.251\| Glycerol-3-phosphate cytidylyltransferase (EC 2.7.7.39) / NAD-dependent oxidoreductase [Helcococcus ovis KG36 \| 72026.48] | 20/37 | 54 | 29/37 | 78 | (hldE) bifunctional D-beta-D-heptose 7-phosphate kinase/D-beta-D-heptose 1-phosphate adenylyltransferase [LOS - Immune modulation] [Campylobacter jejuni subsp. jejuni NCTC 11168] Length = 461 Score = 48.1 bits (113), Expect = 2e-06 Identities = 20/37 (54%), Positives = 29/37 (78%) |
| fig\|72026.48.peg.258\| hypothetical protein [Helcococcus ovis KG36 \| 72026.48] | 81/155 | 52 | 109/155 | 70 | (pspA) surface protein A [PspA - Immune modulation] [Streptococcus pneumoniae TIGR4] Length = 744 Score = 201 bits (510), Expect = 1e-52 Identities = 81/155 (52%), Positives = 109/155 (70%), Gaps = 1/155 (0%) |
| fig\|72026.48.peg.266\| UDP-glucose 4-epimerase (EC 5.1.3.2) [Helcococcus ovis KG36 \| 72026.48] | 196/336 | 58 | 242/336 | 72 | (galE) UDP-glucose 4-epimerase [LOS - Immune modulation] [Haemophilus influenzae Rd KW20] Length = 338 Score = 398 bits (1022), Expect = e-112 Identities = 196/336 (58%), Positives = 242/336 (72%) |
| fig\|72026.48.peg.280\| Choline binding protein A [Helcococcus ovis KG36 \| 72026.48] | 25/51 | 49 | 36/51 | 70 | (pspA) surface protein A [PspA - Immune modulation] [Streptococcus pneumoniae TIGR4] Length = 744 Score = 66.6 bits (161), Expect = 3e-12 Identities = 34/87 (39%), Positives = 48/87 (55%), Gaps = 3/87 (3%) |
| fig\|72026.48.peg.413\| Translation elongation factor Tu [Helcococcus ovis KG36 \| 72026.48] | 290/397 | 73 | 338/397 | 85 | (tufA) elongation factor Tu [EF-Tu - Adherence] [Francisella tularensis subsp. tularensis SCHU S4] Length = 394 Score = 590 bits (1522), Expect = e-170 Identities = 290/397 (73%), Positives = 338/397 (85%), Gaps = 3/397 (0%) |
| fig\|72026.48.peg.450\| Zinc ABC transporter, permease protein ZnuB [Helcococcus ovis KG36 \| 72026.48] | 14/26 | 53 | 20/26 | 76 | (mntB) Manganese transport system membrane protein MntB [MntABC - Stress survival] [Neisseria meningitidis MC58] Length = 291 Score = 77.4 bits (189), Expect = 1e-15 Identities = 70/267 (26%), Positives = 115/267 (43%), Gaps = 14/267 (5%) |
| fig\|72026.48.peg.60\| ATP-dependent Clp protease proteolytic subunit ClpP (EC 3.4.21.92) [Helcococcus ovis KG36 \| 72026.48] | 125/190 | 65 | 159/190 | 83 | (clpP) ATP-dependent Clp protease proteolytic subunit [ClpP - Stress survival] [Listeria monocytogenes EGD-e] |
| fig\|72026.48.peg.642\| hypothetical protein [Helcococcus ovis KG36 \| 72026.48] | 16/27 | 59 | 21/27 | 77 | (clfB) Clumping factor B, adhesin [Clumping factor - Adherence] [Staphylococcus aureus subsp. aureus MW2] Length = 907 Score = 37.0 bits (84), Expect = 0.004 Identities = 16/27 (59%), Positives = 21/27 (77%) |
| fig\|72026.48.peg.914\| Heterodimeric efflux ABC transporter, permease/ATP-binding subunit 1 [Helcococcus ovis KG36 \| 72026.48] | 22/56 | 39 | 41/56 | 73 | (msbA) lipid transporter ATP-binding/permease [LOS - Immune modulation] [Haemophilus influenzae Rd KW20] Length = 587 Score = 235 bits (599), Expect = 9e-63 Identities = 158/565 (27%), Positives = 296/565 (52%), Gaps = 22/565 (3%) |
| fig\|72026.49.peg.1106\| ABC transporter ATP-binding protein [Helcococcus ovis KG37 \| 72026.49] | 16/31 | 51 | 22/31 | 70 | (cylB) ABC-type transporter [Cytolysin - Exotoxin] [Enterococcus faecalis str. MMH594] Length = 714 Score = 33.1 bits (74), Expect = 0.005 Identities = 16/31 (51%), Positives = 22/31 (70%), Gaps = 1/31 (3%) |
| fig\|72026.49.peg.1148\| Type II/IV secretion system ATP hydrolase TadA/VirB11/CpaF, TadA subfamily [Helcococcus ovis KG37 \| 72026.49] | 199/400 | 49 | 281/400 | 70 | (VV1_RS15610) CpaF family protein [Flp pili - Adherence] [Vibrio vulnificus CMCP6] Length = 421 Score = 386 bits (992), Expect = e-108 Identities = 199/400 (49%), Positives = 281/400 (70%), Gaps = 3/400 (0%) |
| fig\|72026.49.peg.119\| Chaperone protein ClpB (ATP-dependent unfoldase) [Helcococcus ovis KG37 \| 72026.49] | 137/248 | 55 | 182/248 | 73 | (clpV1) type VI secretion system AAA+ family ATPase [HSI-1 - Effector delivery system] [Pseudomonas aeruginosa PAO1] Length = 902 Score = 268 bits (686), Expect = 1e-72 Identities = 137/248 (55%), Positives = 182/248 (73%), Gaps = 2/248 (0%) |
| fig\|72026.49.peg.1339\| Heat shock protein 60 kDa family chaperone GroEL [Helcococcus ovis KG37 \| 72026.49] | 335/538 | 62 | 429/538 | 79 | (groEL) chaperonin GroEL [GroEL - Adherence] [Clostridium difficile 630] Length = 542 Score = 648 bits (1672), Expect = 0.0 Identities = 335/538 (62%), Positives = 429/538 (79%), Gaps = 2/538 (0%) |
| fig\|72026.49.peg.1361\| RNA polymerase sigma factor RpoD [Helcococcus ovis KG37 \| 72026.49] | 153/243 | 62 | 199/243 | 81 | (sigA/rpoV) RNA polymerase sigma factor SigA [SigA - Regulation] [Mycobacterium tuberculosis H37Rv] Length = 528 Score = 317 bits (812), Expect = 2e-87 Identities = 153/243 (62%), Positives = 199/243 (81%) |
| fig\|72026.49.peg.1380\| Acetaldehyde dehydrogenase (EC 1.2.1.10) / Alcohol dehydrogenase (EC 1.1.1.1) [Helcococcus ovis KG37 \| 72026.49] | 538/863 | 62 | 675/863 | 78 | (lap) Listeria adhesion protein Lap [Lap - Adherence] [Listeria monocytogenes EGD-e] Length = 866 Score = 1093 bits (2826), Expect = 0.0 Identities = 538/863 (62%), Positives = 675/863 (78%), Gaps = 2/863 (0%) |
| fig\|72026.49.peg.1453\| ABC transporter, ATP-binding protein (cluster 3, basic aa/glutamine/opines) [Helcococcus ovis KG37 \| 72026.49] | 115/221 | 52 | 156/221 | 70 | (bauE) ferric siderophore ABC transporter, ATP-binding protein BauE [Acinetobactin - Nutritional/Metabolic factor] [Acinetobacter baumannii ACICU] Length = 256 Score = 94.0 bits (232), Expect = 1e-20 Identities = 64/221 (28%), Positives = 115/221 (52%), Gaps = 14/221 (6%) |
| fig\|72026.49.peg.1509\| Sortase A, LPXTG specific [Helcococcus ovis KG37 \| 72026.49] | 138/232 | 59 | 188/232 | 81 | (srtC-2/srtC) sortase [RlrA islet - Adherence] [Streptococcus pneumoniae TIGR4] Length = 297 Score = 303 bits (776), Expect = 1e-83 Identities = 138/232 (59%), Positives = 188/232 (81%) |
| fig\|72026.49.peg.1510\| Sortase A, LPXTG specific [Helcococcus ovis KG37 \| 72026.49] | 158/219 | 72 | 186/219 | 84 | (srtC-1/srtB) sortase [RlrA islet - Adherence] [Streptococcus pneumoniae TIGR4] Length = 300 Score = 332 bits (851), Expect = 3e-92 Identities = 158/219 (72%), Positives = 186/219 (84%) |
| fig\|72026.49.peg.1540\| Peptide-methionine (S)-S-oxide reductase MsrA (EC 1.8.4.11) / Peptide-methionine (R)-S-oxide reductase MsrB (EC 1.8.4.12) [Helcococcus ovis KG37 \| 72026.49] | 197/324 | 60 | 237/324 | 73 | (msrA/B(pilB)) trifunctional thioredoxin/methionine sulfoxide reductase A/B protein [MsrAB - Stress survival] [Neisseria meningitidis MC58] Length = 522 Score = 407 bits (1046), Expect = e-115 Identities = 197/324 (60%), Positives = 237/324 (73%), Gaps = 12/324 (3%) |
| fig\|72026.49.peg.254\| Glycerol-3-phosphate cytidylyltransferase (EC 2.7.7.39) / NAD-dependent oxidoreductase [Helcococcus ovis KG37 \| 72026.49] | 20/37 | 54 | 29/37 | 78 | (hldE) bifunctional D-beta-D-heptose 7-phosphate kinase/D-beta-D-heptose 1-phosphate adenylyltransferase [LOS - Immune modulation] [Campylobacter jejuni subsp. jejuni NCTC 11168] Length = 461 Score = 48.1 bits (113), Expect = 2e-06 Identities = 20/37 (54%), Positives = 29/37 (78%) |
| fig\|72026.49.peg.267\| UDP-glucose 4-epimerase (EC 5.1.3.2) [Helcococcus ovis KG37 \| 72026.49] | 196/336 | 58 | 242/336 | 72 | (galE) UDP-glucose 4-epimerase [LOS - Immune modulation] [Haemophilus influenzae Rd KW20] Length = 338 Score = 398 bits (1022), Expect = e-112 Identities = 196/336 (58%), Positives = 242/336 (72%) |
| fig\|72026.49.peg.416\| Translation elongation factor Tu [Helcococcus ovis KG37 \| 72026.49] | 290/397 | 73 | 338/397 | 85 | (tufA) elongation factor Tu [EF-Tu - Adherence] [Francisella tularensis subsp. tularensis SCHU S4] Length = 394 Score = 590 bits (1522), Expect = e-170 Identities = 290/397 (73%), Positives = 338/397 (85%), Gaps = 3/397 (0%) |
| fig\|72026.49.peg.475\| Exodeoxyribonuclease III (EC 3.1.11.2) [Helcococcus ovis KG37 \| 72026.49] | 130/252 | 51 | 177/252 | 70 | (crc) catabolite repression control protein [Type IV pili - Adherence] [Pseudomonas aeruginosa PAO1] Length = 259 Score = 129 bits (323), Expect = 3e-31 Identities = 76/252 (30%), Positives = 130/252 (51%), Gaps = 6/252 (2%) |
| fig\|72026.49.peg.554\| Carbonic anhydrase-like protein MJ0304 [Helcococcus ovis KG37 \| 72026.49] | 22/56 | 39 | 41/56 | 73 | (cap8J) type 8 capsular polysaccharide synthesis protein Cap8J [Capsule - Immune modulation] [Staphylococcus aureus subsp. aureus MW2] Length = 185 Score = 45.8 bits (107), Expect = 4e-06 Identities = 22/56 (39%), Positives = 41/56 (73%), Gaps = 3/56 (5%) |
| fig\|72026.49.peg.618\| Replication-associated recombination protein RarA [Helcococcus ovis KG37 \| 72026.49] | 14/26 | 53 | 20/26 | 76 | (eccA3) Type VII secretion system protein EccA3 [ESX-3 - Effector delivery system] [Mycobacterium tuberculosis H37Rv] Length = 631 Score = 36.2 bits (82), Expect = 0.006 Identities = 14/26 (53%), Positives = 20/26 (76%) |
| fig\|72026.49.peg.62\| ATP-dependent Clp protease proteolytic subunit ClpP (EC 3.4.21.92) [Helcococcus ovis KG37 \| 72026.49] | 125/190 | 65 | 159/190 | 83 | (clpP) ATP-dependent Clp protease proteolytic subunit [ClpP - Stress survival] [Listeria monocytogenes EGD-e] Length = 198 Score = 256 bits (654), Expect = 1e-69 Identities = 125/190 (65%), Positives = 159/190 (83%) |
| fig\|72026.49.peg.646\| hypothetical protein [Helcococcus ovis KG37 \| 72026.49] | 19/36 | 52 | 28/36 | 77 | (esp) Enterococcal surface protein; Esp [Esp - Adherence] [Enterococcus faecalis str. MMH594] Length = 1873 Score = 43.1 bits (100), Expect = 5e-05 Identities = 19/36 (52%), Positives = 28/36 (77%) |
| fig\|72026.49.peg.261\| hypothetical protein [Helcococcus ovis KG37 \| 72026.49] | 85/161 | 52 | 113/161 | 70 | (cbpA/pspC) choline binding protein A [CbpA/PspC - Adherence] [Streptococcus pneumoniae TIGR4] Length = 693 Score = 209 bits (531), Expect = 5e-55 Identities = 85/161 (52%), Positives = 113/161 (70%), Gaps = 1/161 (0%) |
| fig\|72026.50.peg.1240\| Ureidoglycolate dehydrogenase (EC 1.1.1.154) [Helcococcus ovis KG38 \| 72026.50] | 194/349 | 55 | 260/349 | 74 | (allD) ureidoglycolate dehydrogenase [Allantion utilization - Nutritional/Metabolic factor] [Klebsiella pneumoniae subsp. pneumoniae NTUH-K2044] Length = 349 Score = 403 bits (1035), Expect = e-113 Identities = 194/349 (55%), Positives = 260/349 (74%) |
| fig\|72026.50.peg.1273\| hypothetical protein [Helcococcus ovis KG38 \| 72026.50] | 35/79 | 44 | 56/79 | 70 | (aatC) ATP-binding protein AatC [Dispersin - Others] [Escherichia coli O44:H18 042] Length = 209 Score = 67.8 bits (164), Expect = 2e-13 Identities = 35/79 (44%), Positives = 56/79 (70%), Gaps = 2/79 (2%) |
| fig\|72026.50.peg.1274\| hypothetical protein [Helcococcus ovis KG38 \| 72026.50] | 22/47 | 46 | 34/47 | 72 | (pvdE) pyoverdine biosynthesis protein PvdE [Pyoverdine - Nutritional/Metabolic factor] [Pseudomonas aeruginosa PAO1] |
| fig\|72026.50.peg.1310\| Translation elongation factor Tu [Helcococcus ovis KG38 \| 72026.50] | 290/397 | 73 | 339/397 | 85 | (tufA) elongation factor Tu [EF-Tu - Adherence] [Francisella tularensis subsp. tularensis SCHU S4] Length = 394 Score = 593 bits (1528), Expect = e-170 Identities = 290/397 (73%), Positives = 339/397 (85%), Gaps = 3/397 (0%) |
| fig\|72026.50.peg.1425\| UDP-N-acetylglucosamine 2-epimerase (EC 5.1.3.14) [Helcococcus ovis KG38 \| 72026.50] | 258/362 | 71 | 315/362 | 87 | (cps4I) capsular polysaccharide biosynthesis protein Cps4I [Capsule - Immune modulation] [Streptococcus pneumoniae TIGR4] Length = 365 Score = 536 bits (1380), Expect = e-153 Identities = 258/362 (71%), Positives = 315/362 (87%) |
| fig\|72026.50.peg.1439\| UDP-glucose 4-epimerase (EC 5.1.3.2) [Helcococcus ovis KG38 \| 72026.50] | 191/338 | 56 | 239/338 | 70 | (galE) UDP-glucose 4-epimerase [LOS - Immune modulation] [Haemophilus influenzae Rd KW20] Length = 338 Score = 389 bits (998), Expect = e-109 Identities = 191/338 (56%), Positives = 239/338 (70%) |
| fig\|72026.50.peg.1587\| Sortase A, LPXTG specific [Helcococcus ovis KG38 \| 72026.50] | 143/232 | 61 | 192/232 | 82 | (srtC-2/srtC) sortase [RlrA islet - Adherence] [Streptococcus pneumoniae TIGR4] Length = 297 Score = 313 bits (803), Expect = 9e-87 Identities = 143/232 (61%), Positives = 192/232 (82%) |
| fig\|72026.50.peg.1588\| Sortase A, LPXTG specific [Helcococcus ovis KG38 \| 72026.50] | 162/247 | 65 | 197/247 | 79 | (srtC-1/srtB) sortase [RlrA islet - Adherence] [Streptococcus pneumoniae TIGR4] Length = 300 Score = 348 bits (893), Expect = 3e-97 Identities = 162/247 (65%), Positives = 197/247 (79%) |
| fig\|72026.50.peg.1614\| Peptide-methionine (S)-S-oxide reductase MsrA (EC 1.8.4.11) / Peptide-methionine (R)-S-oxide reductase MsrB (EC 1.8.4.12) [Helcococcus ovis KG38 \| 72026.50] | 211/343 | 61 | 256/343 | 74 | (msrA/B(pilB)) trifunctional thioredoxin/methionine sulfoxide reductase A/B protein [MsrAB - Stress survival] [Neisseria meningitidis MC58] Length = 522 Score = 432 bits (1111), Expect = e-122 Identities = 211/343 (61%), Positives = 256/343 (74%), Gaps = 12/343 (3%) |
| fig\|72026.50.peg.1683\| Chaperone protein ClpB (ATP-dependent unfoldase) [Helcococcus ovis KG38 \| 72026.50] | 178/334 | 53 | 240/334 | 71 | (clpC) endopeptidase Clp ATP-binding chain C [ClpC - Stress survival] [Listeria monocytogenes EGD-e] Length = 820 Score = 357 bits (915), Expect = 3e-99 Identities = 178/334 (53%), Positives = 240/334 (71%), Gaps = 6/334 (1%) |
| fig\|72026.50.peg.306\| Acetaldehyde dehydrogenase (EC 1.2.1.10) / Alcohol dehydrogenase (EC 1.1.1.1) [Helcococcus ovis KG38 \| 72026.50] | 538/863 | 62 | 676/863 | 78 | (lap) Listeria adhesion protein Lap [Lap - Adherence] [Listeria monocytogenes EGD-e] Length = 866 Score = 1089 bits (2816), Expect = 0.0 Identities = 538/863 (62%), Positives = 676/863 (78%), Gaps = 2/863 (0%) |
| fig\|72026.50.peg.325\| RNA polymerase sigma factor RpoD [Helcococcus ovis KG38 \| 72026.50] | 152/243 | 62 | 198/243 | 81 | (sigA/rpoV) RNA polymerase sigma factor SigA [SigA - Regulation] [Mycobacterium tuberculosis H37Rv] Length = 528 Score = 315 bits (806), Expect = 1e-86 Identities = 152/243 (62%), Positives = 198/243 (81%) |
| fig\|72026.50.peg.354\| Heat shock protein 60 kDa family chaperone GroEL [Helcococcus ovis KG38 \| 72026.50] | 326/522 | 62 | 413/522 | 79 | (groEL) chaperonin GroEL [GroEL - Adherence] [Clostridium difficile 630] Length = 542 Score = 635 bits (1638), Expect = 0.0 Identities = 326/522 (62%), Positives = 413/522 (79%) |
| fig\|72026.50.peg.650\| Carbonic anhydrase-like protein MJ0304 [Helcococcus ovis KG38 \| 72026.50] | 24/56 | 42 | 43/56 | 76 | (cap8J) type 8 capsular polysaccharide synthesis protein Cap8J [Capsule - Immune modulation] [Staphylococcus aureus subsp. aureus MW2] Length = 185 Score = 49.7 bits (117), Expect = 3e-07 Identities = 24/56 (42%), Positives = 43/56 (76%), Gaps = 3/56 (5%) |
| fig\|72026.50.peg.720\| Replication-associated recombination protein RarA [Helcococcus ovis KG38 \| 72026.50] | 14/26 | 53 | 20/26 | 76 | (eccA3) Type VII secretion system protein EccA3 [ESX-3 - Effector delivery system] [Mycobacterium tuberculosis H37Rv] Length = 631 Score = 36.6 bits (83), Expect = 0.005 Identities = 14/26 (53%), Positives = 20/26 (76%) |
| fig\|72026.50.peg.78\| ATP-dependent Clp protease proteolytic subunit ClpP (EC 3.4.21.92) [Helcococcus ovis KG38 \| | 124/190 | 65 | 159/190 | 83 | (clpP) ATP-dependent Clp protease proteolytic subunit [ClpP - Stress survival] [Listeria monocytogenes EGD-e] Length = 198 Score = 255 bits (652), Expect = 2e-69 Identities = 124/190 (65%), Positives = 159/190 (83%) |
| fig\|72026.50.peg.973\| hypothetical protein [Helcococcus ovis KG38 \| 72026.50] | 16/42 | 38 | 30/42 | 71 | (p216) protein P216, cilium adhesin p97 paralog [P97/P102 paralog family - Adherence] [Mycoplasma hyopneumoniae 232] Length = 1879 Score = 36.6 bits (83), Expect = 0.007 Identities = 16/42 (38%), Positives = 30/42 (71%), Gaps = 2/42 (4%) |
| fig\|72026.51.peg.751\| hypothetical protein [Helcococcus ovis KG104 \| 72026.51] | 30/63 | 47 | 45/63 | 71 | (cbpA/pspC) choline binding protein A [CbpA/PspC - Adherence] [Streptococcus pneumoniae TIGR4] |
| fig\|72026.51.peg.704\| Ribosome small subunit biogenesis RbfA-release protein RsgA [Helcococcus ovis KG104 \| 72026.51] (292 letters) | 19/41 | 46 | 32/41 | 78 | (iroC) ABC transporter [Sal - Nutritional/Metabolic factor] [Klebsiella pneumoniae subsp. pneumoniae NTUH-K2044] Length = 1214 Score = 35.0 bits (79), Expect = 0.008 Identities = 19/41 (46%), Positives = 22/41 (53%) |
| fig\|72026.51.peg.648\| hypothetical protein [Helcococcus ovis KG104 \| 72026.51] | 18/30 | 60 | 25/30 | 83 | (esp) Enterococcal surface protein; Esp [Esp - Adherence] [Enterococcus faecalis str. MMH594] |
| fig\|72026.51.peg.620\| Replication-associated recombination protein RarA [Helcococcus ovis KG104 \| 72026.51] | 14/26 | 53 | 20/26 | 76 | (eccA3) Type VII secretion system protein EccA3 [ESX-3 - Effector delivery system] [Mycobacterium tuberculosis H37Rv] Length = 631 Score = 36.2 bits (82), Expect = 0.006 Identities = 14/26 (53%), Positives = 20/26 (76%) |
| fig\|72026.51.peg.62\| ATP-dependent Clp protease proteolytic subunit ClpP (EC 3.4.21.92) [Helcococcus ovis KG104 \| 72026.51] | 125/190 | 65 | 159/190 | 83 | (clpP) ATP-dependent Clp protease proteolytic subunit [ClpP - Stress survival] [Listeria monocytogenes EGD-e] Length = 198 Score = 256 bits (654), Expect = 1e-69 Identities = 125/190 (65%), Positives = 159/190 (83%) |
| fig\|72026.51.peg.556\| Carbonic anhydrase-like protein MJ0304 [Helcococcus ovis KG104 \| 72026.51] | 22/56 | 39 | 41/56 | 73 | (cap8J) type 8 capsular polysaccharide synthesis protein Cap8J [Capsule - Immune modulation] [Staphylococcus aureus subsp. aureus MW2] Length = 185 Score = 45.8 bits (107), Expect = 4e-06 Identities = 22/56 (39%), Positives = 41/56 (73%), Gaps = 3/56 (5%) |
| fig\|72026.51.peg.414\| Translation elongation factor Tu [Helcococcus ovis KG104 \| 72026.51] | 290/397 | 73 | 338/397 | 85 | (tufA) elongation factor Tu [EF-Tu - Adherence] [Francisella tularensis subsp. tularensis SCHU S4] Length = 394 Score = 590 bits (1522), Expect = e-170 Identities = 290/397 (73%), Positives = 338/397 (85%), Gaps = 3/397 (0%) |
| fig\|72026.51.peg.282\| Choline binding protein A [Helcococcus ovis KG104 \| 72026.51] | 25/51 | 49 | 36/51 | 70 | (toxA) toxin A [TcdA - Exotoxin] [Clostridium difficile 630] Length = 2710 Score = 53.5 bits (127), Expect = 3e-08 Identities = 25/51 (49%), Positives = 36/51 (70%), Gaps = 2/51 (3%) |
| fig\|72026.51.peg.268\| UDP-glucose 4-epimerase (EC 5.1.3.2) [Helcococcus ovis KG104 \| 72026.51] | 196/336 | 58 | 242/336 | 72 | (galE) UDP-glucose 4-epimerase [LOS - Immune modulation] [Haemophilus influenzae Rd KW20] Length = 338 Score = 398 bits (1022), Expect = e-112 Identities = 196/336 (58%), Positives = 242/336 (72%) |
| fig\|72026.51.peg.256\| Glycerol-3-phosphate cytidylyltransferase (EC 2.7.7.39) / NAD-dependent oxidoreductase [Helcococcus ovis KG104 \| 72026.51] | 21/38 | 55 | 27/38 | 71 | (rfaE) ADP-heptose synthase [LOS - Immune modulation] [Haemophilus influenzae Rd KW20] Length = 476 Score = 44.3 bits (103), Expect = 2e-05 Identities = 21/38 (55%), Positives = 27/38 (71%) |
| fig\|72026.51.peg.1606\| Peptide-methionine (S)-S-oxide reductase MsrA (EC 1.8.4.11) / Peptide-methionine (R)-S-oxide reductase MsrB (EC 1.8.4.12) [Helcococcus ovis KG104 \| 72026.51] | 197/324 | 60 | 236/324 | 72 | (msrA/B(pilB)) trifunctional thioredoxin/methionine sulfoxide reductase A/B protein [MsrAB - Stress survival] [Neisseria meningitidis MC58] Length = 522 Score = 405 bits (1042), Expect = e-114 Identities = 197/324 (60%), Positives = 236/324 (72%), Gaps = 12/324 (3%) |
| fig\|72026.51.peg.1575\| Sortase A, LPXTG specific [Helcococcus ovis KG104 \| 72026.51] | 158/219 | 72 | 186/219 | 84 | (srtC-1/srtB) sortase [RlrA islet - Adherence] [Streptococcus pneumoniae TIGR4] Length = 300 Score = 332 bits (851), Expect = 3e-92 Identities = 158/219 (72%), Positives = 186/219 (84%) |
| fig\|72026.51.peg.1574\| Sortase A, LPXTG specific [Helcococcus ovis KG104 \| 72026.51] | 138/232 | 59 | 187/232 | 80 | (srtC-2/srtC) sortase [RlrA islet - Adherence] [Streptococcus pneumoniae TIGR4] Length = 297 Score = 301 bits (772), Expect = 3e-83 Identities = 138/232 (59%), Positives = 187/232 (80%) |
| fig\|72026.51.peg.1457\| Mg(2+) transport ATPase protein C [Helcococcus ovis KG104 \| 72026.51] | 13/37 | 35 | 26/37 | 70 | (mgtC) Mg2+ transport protein [MgtBC - Nutritional/Metabolic factor] [Salmonella enterica subsp. enterica serovar Typhimurium str. LT2] Length = 231 Score = 33.5 bits (75), Expect = 0.004 Identities = 13/37 (35%), Positives = 26/37 (70%) |
| fig\|72026.51.peg.1435\| Acetaldehyde dehydrogenase (EC 1.2.1.10) / Alcohol dehydrogenase (EC 1.1.1.1) [Helcococcus ovis KG104 \| 72026.51] | 538/863 | 62 | 676/863 | 78 | (lap) Listeria adhesion protein Lap [Lap - Adherence] [Listeria monocytogenes EGD-e] Length = 866 Score = 1093 bits (2828), Expect = 0.0 Identities = 538/863 (62%), Positives = 676/863 (78%), Gaps = 2/863 (0%) |
| fig\|72026.51.peg.1415\| RNA polymerase sigma factor RpoD [Helcococcus ovis KG104 \| 72026.51] | 153/243 | 62 | 199/243 | 81 | (sigA/rpoV) RNA polymerase sigma factor SigA [SigA - Regulation] [Mycobacterium tuberculosis H37Rv] Length = 528 Score = 317 bits (812), Expect = 2e-87 Identities = 153/243 (62%), Positives = 199/243 (81%) |
| fig\|72026.51.peg.1390\| Heat shock protein 60 kDa family chaperone GroEL [Helcococcus ovis KG104 \| 72026.51] | 335/538 | 62 | 428/538 | 79 | (groEL) chaperonin GroEL [GroEL - Adherence] [Clostridium difficile 630] Length = 542 Score = 646 bits (1667), Expect = 0.0 Identities = 335/538 (62%), Positives = 428/538 (79%), Gaps = 2/538 (0%) |
| fig\|72026.51.peg.119\| Chaperone protein ClpB (ATP-dependent unfoldase) [Helcococcus ovis KG104 \| 72026.51] | 137/248 | 55 | 182/248 | 73 | (clpV/tssH) type VI secretion system ATPase TssH [T6SS - Effector delivery system] [Klebsiella pneumoniae subsp. pneumoniae NTUH-K2044] Score = 268 bits (686), Expect = 1e-72 Identities = 137/248 (55%), Positives = 182/248 (73%), Gaps = 2/248 (0%) |
| fig\|72026.51.peg.1173\| Type II/IV secretion system ATP hydrolase TadA/VirB11/CpaF, TadA subfamily [Helcococcus ovis KG104 \| 72026.51] | 199/400 | 49 | 281/400 | 70 | (VV1_RS15610) CpaF family protein [Flp pili - Adherence] [Vibrio vulnificus CMCP6] Length = 421 Score = 387 bits (993), Expect = e-108 Identities = 199/400 (49%), Positives = 281/400 (70%), Gaps = 3/400 (0%) |
| fig\|72026.51.peg.1125\| ABC transporter ATP-binding protein [Helcococcus ovis KG104 \| 72026.51] | 16/31 | 51 | 22/31 | 70 | (cylB) ABC-type transporter [Cytolysin - Exotoxin] [Enterococcus faecalis str. MMH594] Length = 714 Score = 33.1 bits (74), Expect = 0.005 Identities = 16/31 (51%), Positives = 22/31 (70%), Gaps = 1/31 (3%) |
| fig\|72026.52.peg.116\| Chaperone protein ClpB (ATP-dependent unfoldase) [Helcococcus ovis KG106 \| 72026.52] | 137/248 | 55 | 182/248 | 73 | (clpC) endopeptidase Clp ATP-binding chain C [ClpC - Stress survival] [Listeria monocytogenes EGD-e] |
| fig\|72026.52.peg.1223\| Type II/IV secretion system ATP hydrolase TadA/VirB11/CpaF, TadA subfamily [Helcococcus ovis KG106 \| 72026.52] | 199/400 | 49 | 281/400 | 70 | (VV1_RS15610) CpaF family protein [Flp pili - Adherence] [Vibrio vulnificus CMCP6] |
| fig\|72026.52.peg.1416\| Heat shock protein 60 kDa family chaperone GroEL [Helcococcus ovis KG106 \| 72026.52] | 335/538 | 62 | 429/538 | 79 | (groEL) chaperonin GroEL [GroEL - Adherence] [Clostridium difficile 630] |
| fig\|72026.52.peg.1436\| RNA polymerase sigma factor RpoD [Helcococcus ovis KG106 \| 72026.52] | 153/243 | 62 | 199/243 | 81 | (sigA/rpoV) RNA polymerase sigma factor SigA [SigA - Regulation] [Mycobacterium tuberculosis H37Rv] |
| fig\|72026.52.peg.1455\| Acetaldehyde dehydrogenase (EC 1.2.1.10) / Alcohol dehydrogenase (EC 1.1.1.1) [Helcococcus ovis KG106 \| 72026.52] | 538/863 | 62 | 675/863 | 78 | (lap) Listeria adhesion protein Lap [Lap - Adherence] [Listeria monocytogenes EGD-e] |
| fig\|72026.52.peg.1603\| Sortase A, LPXTG specific [Helcococcus ovis KG106 \| 72026.52] | 129/244 | 52 | 175/244 | 71 | (srtC-2/srtC) sortase [RlrA islet - Adherence] [Streptococcus pneumoniae TIGR4] |
| fig\|72026.52.peg.1604\| Sortase A, LPXTG specific [Helcococcus ovis KG106 \| 72026.52] | 115/221 | 52 | 156/221 | 70 | (srtC-1/srtB) sortase [RlrA islet - Adherence] [Streptococcus pneumoniae TIGR4] |
| fig\|72026.52.peg.1635\| Peptide-methionine (S)-S-oxide reductase MsrA (EC 1.8.4.11) / Peptide-methionine (R)-S-oxide reductase MsrB (EC 1.8.4.12) [Helcococcus ovis KG106 \| 72026.52] | 198/324 | 61 | 237/324 | 73 | (msrA/B(pilB)) trifunctional thioredoxin/methionine sulfoxide reductase A/B protein [MsrAB - Stress survival] [Neisseria meningitidis MC58] |
| fig\|72026.52.peg.255\| Glycerol-3-phosphate cytidylyltransferase (EC 2.7.7.39) / NAD-dependent oxidoreductase [Helcococcus ovis KG106 \| 72026.52] | 21/38 | 55 | 27/38 | 71 | (hldE) bifunctional D-beta-D-heptose 7-phosphate kinase/D-beta-D-heptose 1-phosphate adenylyltransferase [LOS - Immune modulation] [Campylobacter jejuni subsp. jejuni NCTC 11168] |
| fig\|72026.52.peg.262\| hypothetical protein [Helcococcus ovis KG106 \| 72026.52] | 81/155 | 52 | 109/155 | 70 | (pspA) surface protein A [PspA - Immune modulation] [Streptococcus pneumoniae TIGR4] |
| fig\|72026.52.peg.271\| UDP-glucose 4-epimerase (EC 5.1.3.2) [Helcococcus ovis KG106 \| 72026.52] | 196/336 | 58 | 242/336 | 72 | (galE) UDP-glucose 4-epimerase [LOS - Immune modulation] [Haemophilus influenzae Rd KW20] |
| fig\|72026.52.peg.415\| Translation elongation factor Tu [Helcococcus ovis KG106 \| 72026.52] | 291/397 | 73 | 339/397 | 85 | (tufA) elongation factor Tu [EF-Tu - Adherence] [Francisella tularensis subsp. tularensis SCHU S4] |
| fig\|72026.52.peg.575\| Carbonic anhydrase-like protein MJ0304 [Helcococcus ovis KG106 \| 72026.52] | 22/56 | 39 | 41/56 | 73 | (ricA) type IV secretion system effector RicA, Rab2 interacting conserved protein A [T4SS secreted effectors - Effector delivery system] [Brucella melitensis bv. 1 str. 16M] |
| fig\|72026.52.peg.59\| ATP-dependent Clp protease proteolytic subunit ClpP (EC 3.4.21.92) [Helcococcus ovis KG106 \| 72026.52] | 125/190 | 65 | 159/190 | 83 | (clpP) ATP-dependent Clp protease proteolytic subunit [ClpP - Stress survival] [Listeria monocytogenes EGD-e] |
| fig\|72026.52.peg.640\| Replication-associated recombination protein RarA [Helcococcus ovis KG106 \| 72026.52] | 14/26 | 53 | 20/26 | 76 | (clpV/tssH) type VI secretion system ATPase TssH [T6SS - Effector delivery system] [Klebsiella pneumoniae subsp. pneumoniae NTUH-K2044] |
| fig\|72026.52.peg.670\| hypothetical protein [Helcococcus ovis KG106 \| 72026.52] | 19/36 | 52 | 28/36 | 77 | VFG001361 (pspA) surface protein A [PspA - Immune modulation] [Streptococcus pneumoniae TIGR4] |
