# Supplementary material for: Sequencing and characterization of Helcococcus ovis: a comprehensive comparative genomic analysis of virulence
Source: BMC Genomics. 2023 Aug 30;24:501. doi: 10.1186/s12864-023-09581-1 (PMC10466703; doi:10.1186/s12864-023-09581-1)
Supplement: Supplementary file 12 — Additional file 12: Supplemental Table 8. List of features of ZnuC orthologs found in Helcococcus ovis genomes. These components of Zinc ABC transporters were used for protein multiple sequence alignment analyses. [file 12864_2023_9581_MOESM12_ESM.docx]

**Supplemental Table 8 -** List of features of ZnuC orthologs found in *Helcococcus ovis* genomes. These components of Zinc ABC transporters were used for protein multiple sequence alignment analyses.

| **Genome** | **Feature Type** | **Start** | **End** | **Length** | **Strand** | **AA Length** | **Product** |
| --- | --- | --- | --- | --- | --- | --- | --- |
| Helcococcus ovis KG36 | CDS | 1174366 | 1175037 | 672 | - | 223 | Zinc ABC transporter, ATP-binding protein ZnuC |
| Helcococcus ovis KG36 | CDS | 492859 | 493530 | 672 | + | 223 | Zinc ABC transporter, ATP-binding protein ZnuC |
| Helcococcus ovis KG36 | CDS | 750108 | 750776 | 669 | + | 222 | Zinc ABC transporter, ATP-binding protein ZnuC |
| Helcococcus ovis KG37 | CDS | 1176976 | 1177647 | 672 | - | 223 | Zinc ABC transporter, ATP-binding protein ZnuC |
| Helcococcus ovis KG37 | CDS | 502558 | 503229 | 672 | + | 223 | Zinc ABC transporter, ATP-binding protein ZnuC |
| Helcococcus ovis KG37 | CDS | 757583 | 758251 | 669 | + | 222 | Zinc ABC transporter, ATP-binding protein ZnuC |
| Helcococcus ovis KG38 | CDS | 1290078 | 1290371 | 294 | - | 97 | hypothetical protein |
| Helcococcus ovis KG38 | CDS | 1290462 | 1290749 | 288 | - | 95 | hypothetical protein |
| Helcococcus ovis KG38 | CDS | 170693 | 171364 | 672 | + | 223 | ABC-type antimicrobial peptide transport system, ATPase component |
| Helcococcus ovis KG38 | CDS | 801063 | 801731 | 669 | + | 222 | Zinc ABC transporter, ATP-binding protein ZnuC |
| Helcococcus ovis KG104 | CDS | 1192043 | 1192714 | 672 | - | 223 | Zinc ABC transporter, ATP-binding protein ZnuC |
| Helcococcus ovis KG104 | CDS | 495850 | 496521 | 672 | + | 223 | Zinc ABC transporter, ATP-binding protein ZnuC |
| Helcococcus ovis KG104 | CDS | 752274 | 752942 | 669 | + | 222 | Zinc ABC transporter, ATP-binding protein ZnuC |
| Helcococcus ovis KG106 | CDS | 1243069 | 1243740 | 672 | - | 223 | Zinc ABC transporter, ATP-binding protein ZnuC |
| Helcococcus ovis KG106 | CDS | 498389 | 499060 | 672 | + | 223 | Zinc ABC transporter, ATP-binding protein ZnuC |
| Helcococcus ovis KG106 | CDS | 773583 | 774251 | 669 | + | 222 | Zinc ABC transporter, ATP-binding protein ZnuC |
