# Supplementary material for: Sequencing and characterization of Helcococcus ovis: a comprehensive comparative genomic analysis of virulence
Source: BMC Genomics. 2023 Aug 30;24:501. doi: 10.1186/s12864-023-09581-1 (PMC10466703; doi:10.1186/s12864-023-09581-1)
Supplement: Supplementary file 13 — Additional file 13: Supplemental Table 9. Accession numbers for nucleotide sequences used in Figure 1 phylogenetic analyses. [file 12864_2023_9581_MOESM13_ESM.docx]

**Supplemental Table 9 –** Accession numbers for nucleotide sequences used in Figure 1 phylogenetic analyses.

| **Strain No.** | **Species** | **Accession number** |
| --- | --- | --- |
| KG36 | *Helcococcus ovis* | SCFS00000000 |
| KG37 | *Helcococcus ovis* | SCFR00000000 |
| KG38 | *Helcococcus ovis* | CP121192 |
| KG104 | *Helcococcus ovis* | CP119762 |
| KG106 | *Helcococcus ovis* | CP119761 |
| 713 | *Helcococcus ovis* | LC367043.1 |
| 1122 | *Helcococcus ovis* | LC367049.1 |
| S840-96-1 | *Helcococcus ovis* | NR_027228.1 |
| H1 | *Helcococcus ovis* | AB542078 |
| Tongji | *Helcococcus ovis* | MG188744 |
| Type | *Helcococcus kunzii* | GCA_000245755.1 |
| Type | *Helcococcus sueciensis* | GCA_000423145.1 |
| Type | *Helcococcus massiliensis* | GCA_900258485.1 |
